# Ferroptosis in Neurons and Cancer Cells Is Similar But Differentially Regulated by Histone Deacetylase Inhibitors

**DOI:** 10.1523/ENEURO.0263-18.2019

**Published:** 2019-02-15

**Authors:** Marietta Zille, Amit Kumar, Nandini Kundu, Megan W. Bourassa, Victor S. C. Wong, Dianna Willis, Saravanan S. Karuppagounder, Rajiv R. Ratan

**Affiliations:** 1 Burke Neurological Institute, White Plains, New York 10605; 2 Feil Family Brain and Mind Research Institute, Weill Cornell Medicine, New York, New York 10065; 3 Fraunhofer Research Institution for Marine Biotechnology and Cell Technology and Institute for Medical and Marine Biotechnology, University of Lübeck, Lübeck, 23562, Germany; 4 Institute for Experimental and Clinical Pharmacology and Toxicology, University of Lübeck, Lübeck, 23562, Germany

**Keywords:** apoptosis, cell death, chemotherapy, ferroptosis, necroptosis, necrosis

## Abstract

Ferroptotic death is a mechanism for tumor suppression by pharmacological inhibitors that target the X_c_^−^ transporter (cystine/glutamate antiporter) in a host of non-CNS and CNS tumors. Inhibition of this transporter leads to reduction of cystine uptake, cyst(e)ine deprivation, subsequent depletion of the versatile antioxidant glutathione, and reactive lipid species-dependent death. Accordingly, pharmacological inhibitors of the X_c_^−^ transporter can also induce neuronal cell death raising concerns about toxicity in the CNS and PNS if these agents are used for chemotherapy. Here, we show that ferroptotic death induced by the canonical ferroptosis inducer erastin is similar in HT1080 fibrosarcoma cells and primary cortical neurons although cell death is mediated more potently in cancer cells. Reducing the toxicity of ferroptosis inducers will require, among other things, the identification of agents that protect neurons from ferroptosis but exacerbate it in tumor cells. Although we show that a number of agents known to block ferroptosis in primary mouse neurons also inhibit ferroptosis in fibrosarcoma cells, class I histone deacetylase (HDAC) inhibitors selectively protect neurons while augmenting ferroptosis in cancer cells. Our results further suggest that cell death pathways induced by erastin in these two cell types are statistically identical to each other and identical to oxidative glutamate toxicity in neurons, where death is also mediated via inhibition of X_c_^−^ cystine transport. Together, these studies identify HDACs inhibitors as a novel class of agents to augment tumor suppression by ferroptosis induction and to minimize neuronal toxicity that could manifest as peripheral neuropathy or chemo brain.

## Visual Abstract

**Figure:**
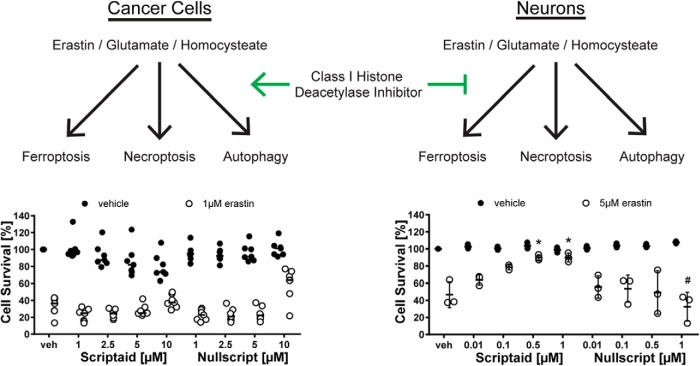

## Significance Statement

A major challenge in cancer chemotherapy is to effectively kill tumor cells while preserving healthy tissue. The nervous system is particularly vulnerable to side effects by anti-cancer agents. Agents that induce a recently identified type of cell death, called ferroptosis, are widely being considered for cancer treatment. However, precise understanding of how ferroptosis induction in cancer cells may simultaneously thwart function or viability of post-mitotic neurons is essential in defining the efficacy and toxicity of these agents. We show that mechanisms of ferroptotic cell death in cancer cells are similar to those in neurons. We leverage prior studies of ferroptosis in neurons to identify histone deacetylase inhibitors as agents that enhance chemotherapy-induced ferroptosis of tumors while inhibiting ferroptosis in neurons.

## Introduction

Cancer is among the leading causes of death worldwide, and its incidence is expected to increase within the next decades. Accordingly, there are intense efforts to develop novel small molecules that can induce death of cancer cells. In an attempt to target tumor cells with activating mutations of RAS, which are present in around a third of all cancers, Dixon et al. (2012) identified the small molecule chemotherapeutic agent erastin. The authors characterized the mechanisms of erastin-induced death and found that cells die in an iron-dependent form of non-apoptotic cell death they called ferroptosis. They further showed that erastin induces ferroptosis by selectively inhibiting the plasma membrane transport of cyst(e)ine via a well-characterized cystine/glutamate exchanger (X_c_^−^). Cyst(e)ine depletion leads to decreased levels of glutathione (Bridges et al., 2012; Lewerenz et al., 2013), culminating in cell death due to the production of reactive lipid species (Tan et al., 1998; Yang and Stockwell, 2016).

A major challenge in effective cancer chemotherapy is to reduce side effects on the central nervous system (e.g., chemo brain-deficits in cognitive function, memory, and attention) or peripheral nervous system (e.g., neuropathic pain) toxicities (Banach et al., 2017; Vitali et al., 2017). These toxicities can reduce the quality of life and functional status even in circumstances where chemotherapy effectively neutralizes the tumor (Kerckhove et al., 2017).

To understand the potential toxicities of erastin in CNS neurons, we here examine the signaling pathways engaged by erastin in post-mitotic cortical neurons and compare them to those activated in HT1080 fibrosarcoma cells.

## Materials and Methods

### Chemicals and reagents

Apicidin (catalog #10575), DPQ (14450), and Nullscript (16433) were obtained from Cayman Chemical. 3-Methyladenine (BML-AP502-0025), Mdivi-1 (BML-CM127-0010), Necrostatin-1 (BML-AP309-0020), Scriptaid (BML-GR326-0005), Trolox (ALX-270-267-M100), and z-VAD-fmk (ALX-260-138-R100) were purchased from Enzo Life Sciences. Bafilomycin A1 (B_1080_), cyclosporine A (C-6000), Olaparib (O-9201), SB203580 (S-3400), SP600125 (S-7979), and U0126 (U-6770) were obtained from LC Laboratories. Laminin (CC095), GSK’872 (530389), Necrostatin-1 inactive (480066), Necrosulfonamide (480073) mouse tumor necrosis factor-α (GF027), U0124 (662006), and rabbit anti-acetylated Histone H4 (1:5000; 06-866; RRID:AB_310270) antibody were from Millipore; Boc-DON-Gln-Ile-Val-OMe (B003), 1,3-dimethyl-4,5-diphenyl-2-[(2-oxopropyl)thio]imidazolium trifluorosulfonic acid salt (D004) from Zedira GmbH; 3-(4,5-dimethylthiazol-2-yl)-2,5-diphenyltetrazolium bromide (MTT assay, G4100) from Promega; erastin (S7242) and entinostat (MS-275, S1053) from Selleck Chemicals. Actinomycin D (A1410), Chloroquine (C6628), cycloheximide (01810), Cystamine dihydrochloride (C8707), Deferoxamine (D9533), Ferrostatin-1 (SML0583), Homocysteate (H9633), Mithramycin A (M6891), *N*-acetylcysteine (A7250), rapamycin (R8781), sodium butyrate (303410), protease inhibitor cocktail (P8340), poly-d-lysine (P6407), poly-l-lysine (P4704), N1 supplement (N6530), collagenase (C9697), EGTA (E0396), sodium orthovanadate (S6508), mouse anti-β-actin (1:20,000; clone AC-74, A5316; RRID:AB_476743), and Tween 20 (P7949) were obtained from Sigma-Aldrich. EDTA (E177) was obtained from Amresco. Triton X-100 (161-0407), DC Protein Assay Kit I (5000111), Quick Start Bradford Reagent (500-0205), and Protein Dual Color Standard (161-0374) were purchased from Bio-Rad. DMEM (11965118), DMEM/nutrient mixture F-12 (10565-018), MEM GlutaMAX Supplement (41090101), MEM non-essential amino acids (11140050), fetal bovine serum (16140071), horse serum (26050088), penicillin-streptomycin (15140163), live/dead assay (L3224), NuPAGE 4% and 12% Bis-Tris protein gels (NP0335 and NP0336), MES SDS Running Buffer (NP0002), TaqMan c-Myc (Hs00153408), p21 (Hs00355782), HDAC1 (Hs00606262_g1), HDAC2 (Hs00231032_m1), HDAC3 (Hs00187320_m1), HDAC8 (Hs00954353_g1), GAPDH (4332649) human primers, HDAC1 (Mm02391771), HDAC2 (Mm00515108), HDAC3 (Mm00515916), HDAC8 (Mm01224980_m1) mouse primers, mouse β-actin endogenous control VIC (4352341E), TaqMan RNA-to-CT 1-Step Kit (4392656), and MicroAmp 96-well Reaction Plates (4346906) were purchased from ThermoFisher Scientific. Laemmli SDS Sample Buffer (BP-110R), Transfer Buffer (BP-190), and Tris-Buffered Saline (BM-300) were obtained from Boston BioProducts. Methanol (BDH1135) was purchased from VWR. Mouse anti-Histone H4 (1:5000; 2960S; RRID:AB_1147657) antibody was from Cell Signaling Technology. Rabbit anti-phospho-S166 RIP1 antibody (1:2000) was provided by P.J.G., J.B., and J.F. (GlaxoSmithKline). Nitrocellulose membrane 0.2 µm (10600001) was from GE Healthcare. Odyssey Blocking Buffer (927-40010), goat anti-rabbit 680RD (1:20,000, 926-68071; RRID:AB_10956166), and goat anti-mouse 800CW (1:20,000; 926-32210; RRID:AB_621842) were purchased from LI-COR Biosciences. NucleoSpin RNA isolation kit (740955) was obtained from Clontech. Eagle’s Minimum Essential Medium (30-2003) was from American type Culture Collection.

3-Methyladenine, actinomycin D, Apicidin, Bafilomycin A1, B003, Cycloheximide, ayclosporine A, D004, DPQ, erastin, ferrostatin-1, Mdivi-1, Mithramycin A, MS-275, Necrostatin-1, Necrostatin-1 inactive, Nullscript, Olaparib, rapamycin, SB203580, Scriptaid, SP600125, U0124, U0126, and z-VAD-fmk were dissolved in DMSO. Chloroquine, Cystamine dihydrochloride, Deferoxamine, and *N*-acetylcysteine were dissolved in water, Tumor necrosis factor-α in PBS, and Trolox in ethanol. Homocysteate was dissolved in MEM and further diluted in water to 250 mM stock solution. Sodium butyrate was dissolved in culture media.

### Animals

All animal procedures were approved by the Weill Cornell Medicine Institutional Animal Care and Use Committee (Approval #0707-633A) and conducted in accordance with the NIH *Guide for the Care and Use of Laboratory Animals* and ARRIVE guidelines. Mice were purchased from Charles River Laboratories and housed at 20–22°C, 30–70% humidity, under a 12 h light/dark cycle, with food (PicoLab Rodent diet 5053, LabDiet) and water *ad libitum*.

### Cell culture

Primary cortical neurons were obtained from CD-1/ICR mice of either sex at embryonic day 14.5. Briefly, cortices were dissected, homogenized, and plated in poly-d-lysine-coated plates in minimum essential medium containing 10% fetal bovine serum, 5% horse serum, and 1% penicillin/streptomycin (1,000,000 cells/ml).

Primary dorsal root ganglia (DRG) neurons were obtained from C57BL/6 mice of either sex at 6 weeks of age. Briefly, the spinal column was isolated by dissection and the spinal cord removed by hydraulic extrusion. The spinal cord was split longitudinally and the DRGs located and removed. DRGs were dissociated with collagenase, plated onto poly-l-lysine/laminin-coated plates in DMEM/nutrient mixture F-12 supplemented with 1× N1 and 10% horse serum (1,000,000 cells/ml).

Immortalized hippocampal neuroblasts (HT22 cells) were cultured in DMEM containing 10% fetal bovine serum and 1% penicillin/streptomycin (50,000 cells/ml). HT1080 cells were obtained from American type Culture Collection and cultured in DMEM containing 10% fetal bovine serum, 1% penicillin/streptomycin, and 1% non-essential amino acids (25,000 cells/ml). Hep3B cells were obtained from American type Culture Collection and cultured in Eagle’s minimum essential medium containing 10% fetal bovine serum and 1% penicillin/streptomycin (50,000 cells/ml). SH-SY5Y cells were also obtained from American type Culture Collection and cultured in DMEM/nutrient mixture F-12 containing 10% fetal bovine serum and 1% penicillin/streptomycin (50,000 cells/ml). Cell lines were treated at 24 h when density reached 70% confluency.

All cells were cultured at 37°C in a humidified 5% CO_2_ atmosphere.

### Cell viability

Cell viability was determined at 22–26 h following erastin or glutamate analog homocysteate (HCA) exposure using MTT assay, a colorimetric assay of cell metabolic activity. We measured the plates at SpectraMax Plus Microplate Reader using SoftMax Pro v4.7.1 (both Molecular Devices). The results of population, quantitative assays of cell viability (MTT) were verified by qualitative LIVE/DEAD assay and fluorescence microscopy at Nikon Eclipse TS100 microscope using Nikon DS-L3 (Nikon Instruments).

### Immunoblot analysis

Protein extracts were prepared using 1% Triton buffer (in mm :25 Tris, pH 7.4, 100 NaCl, 1 EGTA, 1% Triton X-100, protease inhibitors, 2.5 sodium orthovanadate) except in case of the quantification of histones, where RIPA-B lysis buffer (1% Triton X-100, 1% SDS, 50 mm Tris-Cl, pH 7.4, 500 mm NaCl, 1 mm EDTA) was used. We electrophoresed the samples under reducing conditions on NuPAGE gels and transferred them to a nitrocellulose membrane. Antibodies against phospho-S166 RIP1, acetylated histone H4, total histone H4, and β-actin were incubated overnight at 4°C. Secondary antibodies were incubated for 1 h at room temperature. We detected the proteins using Odyssey infrared imaging system (LI-COR Biosciences).

### RNA extraction and real-time PCR

The total RNA was prepared using the NucleoSpin RNA isolation kit according to established protocols. We performed real-time PCR using TaqMan RNA-to-CT 1-Step Kit for human c-Myc (Hs00153408), p21 (Hs00355782), HDAC1 (Hs00606262_g1), HDAC2 (Hs00231032_m1), HDAC3 (Hs00187320_m1), HDAC8 (Hs00954353_g1), and mouse HDAC1 (Mm02391771), HDAC2 (Mm00515108), HDAC3 (Mm00515916), and HDAC8 (Mm01224980_m1) at a 7500 Real-Time PCR System (Applied Biosystems). Expression levels were normalized to mouse β-actin endogenous control.

### Statistical analysis

**Figure 1.**
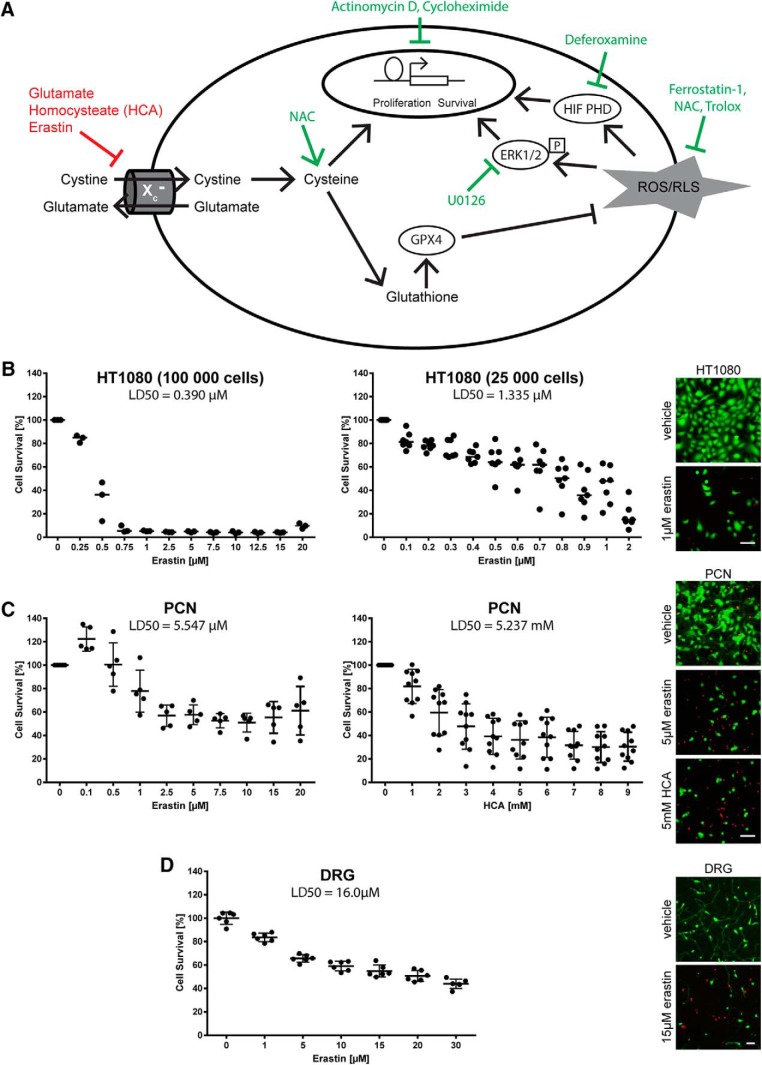
Models of cysteine deprivation. ***A***, The ferroptosis inhibitor erastin is a selective inhibitor of the X_c−_ transporter. Similarly, in cells devoid of ionotropic glutamate receptors, such as tumor cells and immature neurons, glutamate and its analog HCA work as nonspecific inhibitors of the System X_c−_ transporter by counteracting the glutamate gradient resulting in reduced uptake of cystine into the cells. This leads to a decrease in glutathione synthesis, which is essential for the endogenous antioxidant defense. Ferroptosis inhibitors are indicated in green. NAC, *N*-acetylcysteine. ***B***, HT1080 fibrosarcoma cells were treated with increasing dose of erastin at previously described density (left; 100,000 cells/ml) and adjusted density to reach 70% confluency before treatment (right; 25,000 cells/ml) to determine the toxicologically meaningful dose of erastin. Representative live/dead staining are shown, green indicating live cells (calcein AM) and red indicating dead cells (ethidium homodimer-1). Scale bar, 50 µm. ***C***, Dose–response of erastin (left) and HCA (right) in primary cortical neurons. Representative live/dead staining are shown. Scale bar, 50 µm. ***D***, Dose–response of erastin in DRG neurons and representative live/dead staining. Scale bar, 100 µm.

**Figure 2.**
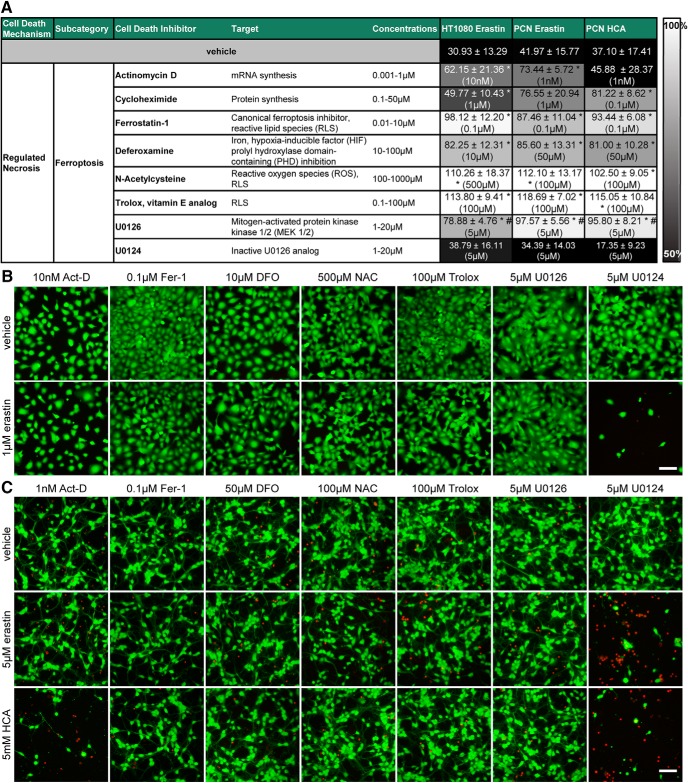
Ferroptosis inhibitors abrogate ferroptosis in cancer cells (HT1080) and primary cortical neurons (PCNs). ***A***, HT1080 cells were treated with 1 µM erastin, PCNs with 5 µM erastin or 5 mM glutamate analog HCA (all LD_50_) glutamate analog HCA and chemical inhibitors effective in ferroptosis were examined. Numbers show mean ± SD at representative concentration in brackets. Grayscale coding indicates the continuum from no protection in the presence of erastin (black) to maximal cell viability (white). *p < 0.05 versus erastin or glutamate analog (HCA), #*p* < 0.05 versus inactive analog U0124. ***B***, ***C***, Representative live/dead staining in HT1080 cells (***B***) and PCN (***C***) are shown, green indicating live cells (calcein AM) and red indicating dead cells (ethidium homodimer-1). Images for cycloheximide are shown in Figure 8 because it is also a criterion for apoptosis. Scale bar, 50 µm.

**Figure 3.**
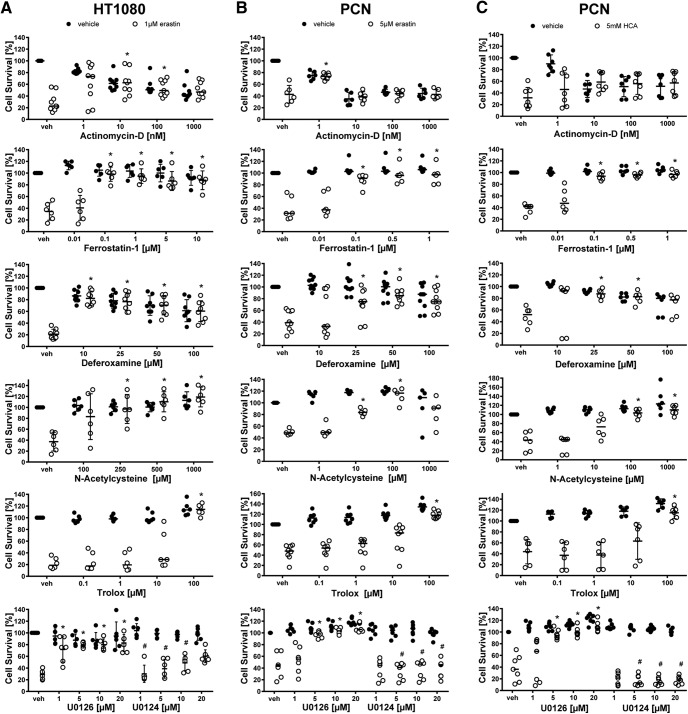
Dose–responses of ferroptosis inhibitors in cancer cells (HT1080) and primary cortical neurons (PCNs). HT1080 cells were treated with 1 µM erastin, PCN with 5 µM erastin or 5 mM glutamate analog HCA and chemical inhibitors effective in ferroptosis were examined. Dose–response for cycloheximide is shown in Figure 9 because it is also a criterion for apoptosis. Values represent mean ± SD, except for actinomycin D and Trolox in HT1080 cells, Ferrostatin-1, Deferoxamine, *N*-acetylcysteine, Trolox, and U0126 in PCN treated with erastin as well as Ferrostatin-1, Deferoxamine, *N*-acetylcysteine, and U0126 in PCNs treated with glutamate analog (HCA) where medians are given. *p < 0.05 versus erastin or glutamate analog (HCA), #*p* < 0.05 versus U0124. For exact *p* values refer to Figure 3-1.

**Figure 4.**
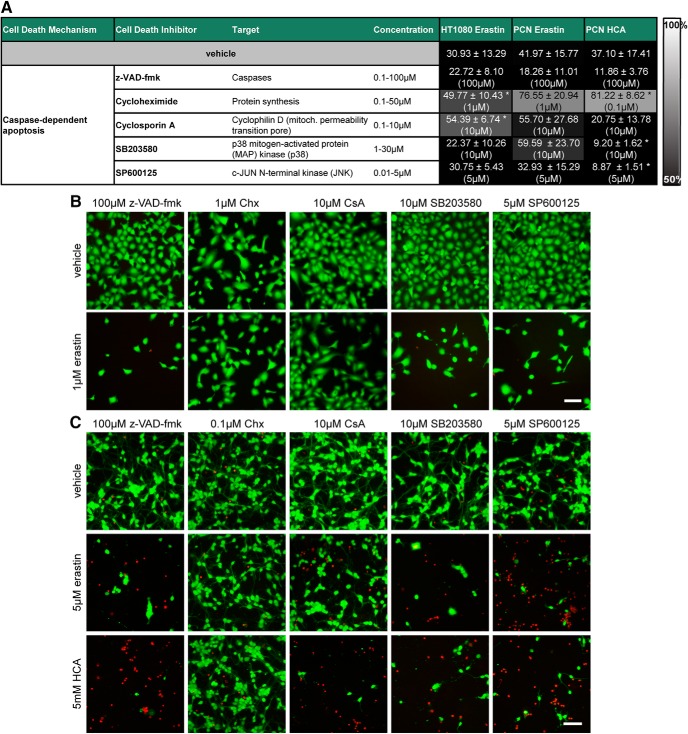
Apoptosis inhibitors do not inhibit ferroptosis in cancer cells (HT1080) and primary cortical neurons (PCNs). ***A***, HT1080 cells were treated with 1 µM erastin, PCN with 5 µM erastin or 5 mM glutamate analog HCA and chemical inhibitors effective in apoptosis were examined. Numbers show mean ± SD at representative concentration in brackets. Grayscale coding indicates the continuum from no protection in the presence of erastin (black) to maximal cell viability (white). *p < 0.05 versus erastin or glutamate analog (HCA). ***B***, ***C***, Representative live/dead staining in HT1080 cells (***B***) and PCNs (***C***) are shown, green indicating live cells (calcein AM) and red indicating dead cells (ethidium homodimer-1). Scale bar, 50 µm.

**Figure 5.**
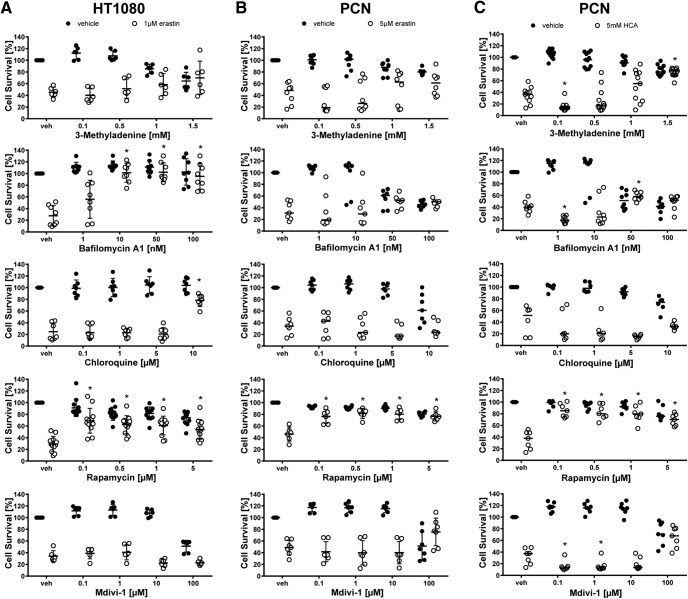
Dose–responses of apoptosis inhibitors in cancer cells (HT1080) and primary cortical neurons (PCNs). HT1080 cells were treated with 1 µM erastin, PCN with 5 µM erastin or 5 mM glutamate analog HCA and chemical inhibitors effective in apoptosis were examined. Values represent mean ± SD, except for cyclosporin A, SP600125, cycloheximide in HT1080 cells, cyclosporin A in PCNs treated with erastin as well as z-VAD-fmk, cyclosporin A, SB203580, SP600125 in PCNs treated with glutamate analog (HCA) where medians are given. *p < 0.05 versus erastin or glutamate analog (HCA). For exact *p* values refer to Figure 5-1.

All data represent biological replicates. For the MTT assay, each biological replicate is the mean of four technical replicates. Normality was evaluated with the Kolmogorov–Smirnov test and variance homogeneity using the Levené test. When data were normally distributed and variance was homogeneous, we performed one-way ANOVA followed by the *post hoc* Bonferroni test. In case one of the criteria was not met, the Kruskal–Wallis test was performed followed by the *post hoc* Mann–Whitney *U* test with α correction according to Bonferroni to adjust for the inflation of type I error due to multiple testing. Data are represented as mean ± SD except for nonparametric data, where medians are given. A value of *p* < 0.05 was considered statistically significant. For the Kruskal–Wallis test followed by Mann–Whitney *U*, *p* = 0.05/*k* was used, with *k* as the number of single hypotheses. *K* = 2 for gene expression experiments (comparison of 2 different concentrations vs vehicle-treated cells), *k* = 4 (comparison of 3 different concentrations vs vehicle-treated cells) for all nonparametric data of drug treatments, except for Necrostatin-1, Scriptaid, and U0126, where *k* = 12 (comparison of 4 different concentrations vs vehicle-treated cells and additional four comparisons vs inactive analog), and pRIP1, where *k* = 9 (all vs 0 h treatment and Necrostatin-1 vs same condition without Necrostatin-1). Thus α = 0.025 for two comparisons, α = 0.0125 for four comparisons, α = 0.0056 for 9 comparisons, and α = 0.0042 for 12 comparisons was considered statistically significant. To analyze contingency tables, Fisher’s exact test was used. Detailed statistical analyses can be found in the extended data (Figs. 3-1, 5-1, 7-1, 9-1, 10-1, 13-1, 13-2, 13-3, 13-4, and 14-1). All statistical analyses were performed with IBM SPSS v23 (RRID:SCR_002865).

10.1523/ENEURO.0263-18.2019.f3-1 Figure 3-1 Statistical data on ferroptosis inhibitors in HT1080 cells and primary cortical neurons. Download Figure 3-1, DOCX file.

10.1523/ENEURO.0263-18.2019.f5-1 Figure 5-1 Statistical data on apoptosis inhibitors in HT1080 cells and primary cortical neurons. Download Figure 5-1, DOCX file.

10.1523/ENEURO.0263-18.2019.f7-1 Figure 7-1 Statistical data on parthanatos and necroptosis inhibitors in HT1080 cells and primary cortical neurons. Download Figure 7-1, DOCX file.

10.1523/ENEURO.0263-18.2019.f9-1 Figure 9-1 Statistical data on autophagy inhibitors in HT1080 cells and primary cortical neurons. Download Figure 9-1, DOCX file.

10.1523/ENEURO.0263-18.2019.f10-1 Figure 10-1 Statistical data on levels of pRIP1 in erastin- and glutamate analog (HCA)-induced cell death. Download Figure 10-1, DOCX file.

10.1523/ENEURO.0263-18.2019.f13-1 Figure 13-1 Statistical data on cell death inhibitors in erastin-induced cell death in HT1080 cells. Download Figure 13-1, DOCX file.

10.1523/ENEURO.0263-18.2019.f13-2 Figure 13-2 Statistical data on gene expression after mithramycin treatment in HT1080 cells. Download Figure 13-2, DOCX file.

10.1523/ENEURO.0263-18.2019.f13-3 Figure 13-3 Statistical data on Scriptaid and Nullscript in erastin-induced death in primary cortical neurons. Download Figure 13-3, DOCX file.

10.1523/ENEURO.0263-18.2019.f13-4 Figure 13-4 Statistical data on HDAC gene expression in primary cortical neurons versus HT1080 cells. Download Figure 13-4, DOCX file.

10.1523/ENEURO.0263-18.2019.f14-1 Figure 14-1 Statistical data on Scriptaid in erastin-induced cell death in SH-SY5Y and Hep3B cells. Download Figure 14-1, DOCX file.

## Results

### Erastin-induced ferroptosis in cancer cells is similar to erastin- and glutamate-induced toxicity in neurons

Ferroptosis has been shown to be induced by cyst(e)ine deprivation (Fig. 1*A*; Bridges et al., 2012; Lewerenz et al., 2013) and is operationally defined by sensitivity to a panel of inhibitors targeting macromolecular synthesis (e.g., cycloheximide), reactive lipids (e.g., ferrostatin-1, *N*-acetylcysteine, Trolox), iron (e.g., DFO), and ERK signaling (e.g., U0126; Dixon et al., 2012, 2014). The initial goal of the current studies was to determine whether erastin-induced ferroptosis in cancer cells occurs via mechanisms that are similar or distinct from those induced by erastin in primary neurons.

Incubation of HT1080 fibrosarcoma cells with increasing concentrations of erastin revealed that doses of erastin used previously (10 µm) to study the mechanisms of death led to a 95% reduction in cell survival (Fig. 1*B*, left), a higher degree of cell death than the LD_50_ usually targeted for toxicological studies. Studies of chemotherapeutic agents at their LD_50_ allows one to discern the protective mechanism from the detrimental effects of the chemical and molecular manipulations of cellular pathways. Accordingly, we adjusted the density of HT1080 cells to 70% confluence before the treatment with different doses of erastin, and found that 1 µM is around the LD_50_ in this particular cell line (Fig. 1*B*, right). In primary neurons, we found the LD_50_ at around 5 µM (Fig. 1*C*, left). Although cortical neuron dysfunction/death may be responsible for “chemo-brain”, DRG sensory neurons dysfunction/death is likely responsible for chemotherapy-induced neuropathy. Of note, we also found that erastin-induced death of DRG sensory neurons (Fig. 1*D*). Because it is better characterized, we focused on erastin-induced death in cortical neurons for the remainder of our studies.

We performed a systematic analysis of ferroptosis inhibitors of erastin-induced death and confirmed that they prevent erastin-induced toxicity in both HT1080 cells and primary neurons (Figs. 2, 3). Moreover, live/dead assays, representing a visible, fluorescence microscopic measure of cell death in single cells, revealed results that were similar to quantitative measurements of cell death performed with MTT assays (Fig. 2).

Interestingly, cyst(e)ine or glutathione depletion has been elucidated as an *in vitro* model of neuronal death in the late 1980s, where glutamate or its analogs were used to induce cell death in cultured neurons (at 2 d *in vitro*) via a non-receptor-mediated mechanism involving inhibition of the System X_c_^−^ (cystine/glutamate) antiporter (Fig. 1*A*). This model has been leveraged to understand how cystine deprivation leads to death via oxidative stress (Murphy et al., 1989; Ratan et al., 1994a, b). To determine whether glutamate analog (HCA) and erastin induce neuronal death via similar pathways, we examined the ability of ferroptosis inhibitors to abrogate glutamate analog (HCA)-induced death. We found that glutamate analog (HCA)-induced death (at the LD_50_ of 5 mm; Fig. 1*C*, right) was abrogated by inhibitors of ferroptosis (Figs. 2, 3).

### Ferroptosis in cancer cells and neurons is abrogated by inhibitors of autophagy and necroptosis

**Figure 6.**
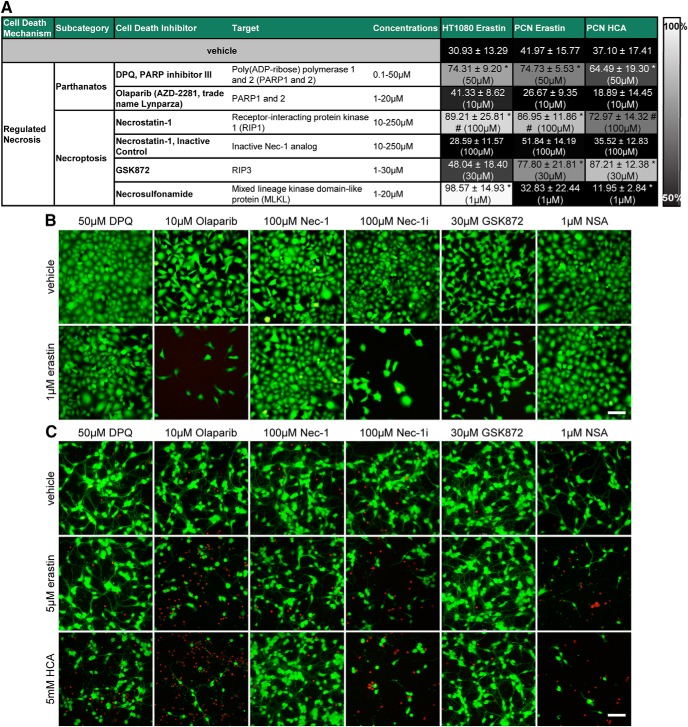
Necroptosis inhibitors, but not parthanatos inhibitors, inhibit ferroptosis in cancer cells (HT1080) and primary cortical neurons (PCNs). ***A***, HT1080 cells were treated with 1 µM erastin, PCNs with 5 µM erastin or 5 mM glutamate analog HCA and chemical inhibitors effective in necroptosis and parthanatos were examined. Numbers show mean ± SD at representative concentration in brackets. Grayscale coding indicates the continuum from no protection in the presence of erastin (black) to maximal cell viability (white). *p < 0.05 versus erastin or glutamate analog (HCA), #*p* < 0.05 versus Necrostatin-1i. ***B***, ***C***, Representative live/dead staining in HT1080 cells (***B***) and PCN (***C***) are shown, green indicating live cells (calcein AM) and red indicating dead cells (ethidium homodimer-1). Scale bar, 50 µm.

**Figure 7.**
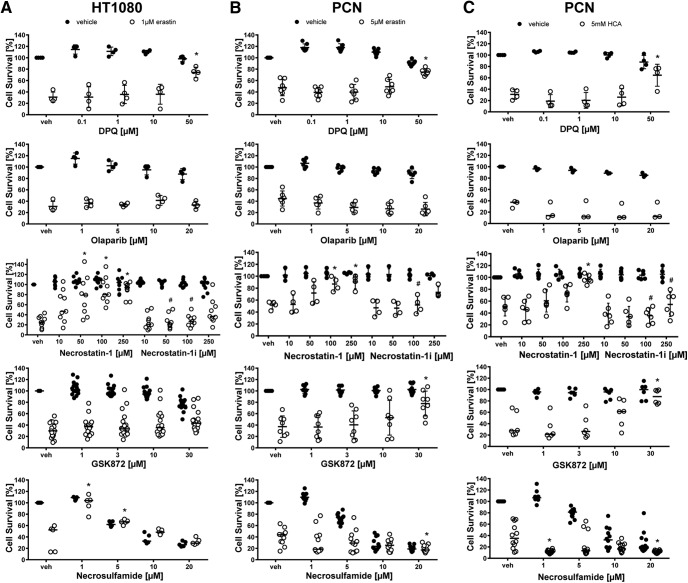
Dose–responses of parthanatos and necroptosis inhibitors in cancer cells (HT1080) and primary cortical neurons (PCNs). HT1080 cells were treated with 1 µM erastin, PCN with 5 µM erastin or 5 mM glutamate analog HCA and chemical inhibitors effective in necroptosis and parthanatos were examined. Values represent mean ± SD, except for Necrostatin-1 and necrosulfonamide in HT1080 cells, necrosulfonamide in PCNs treated with erastin as well as Olaparib, GSK872, and necrosulfonamide in PCNs treated with glutamate analog (HCA) where medians are given. *p < 0.05 versus erastin or glutamate analog (HCA), #*p* < 0.05 versus Necrostatin-1i. For exact *p* values refer to Figure 7-1.

There are many other modes of cell death (Galluzzi et al., 2018). Apoptosis is a caspase-dependent mode of regulated cell death initiated by perturbations of the intracellular (intrinsic apoptosis) or extracellular (extrinsic apoptosis) microenvironment, whereas parthanatos is pathway-dependent on apoptosis-inducing factor that is induced by poly(ADP-ribose) polymerase 1 hyperactivation. We found that erastin-induced death of HT1080 fibrosarcoma cells or primary neurons, as well as glutamate analog (HCA)-induced death of primary neurons, was not altered by inhibitors of apoptosis or parthanatos (Figs. 4–7).

**Figure 8.**
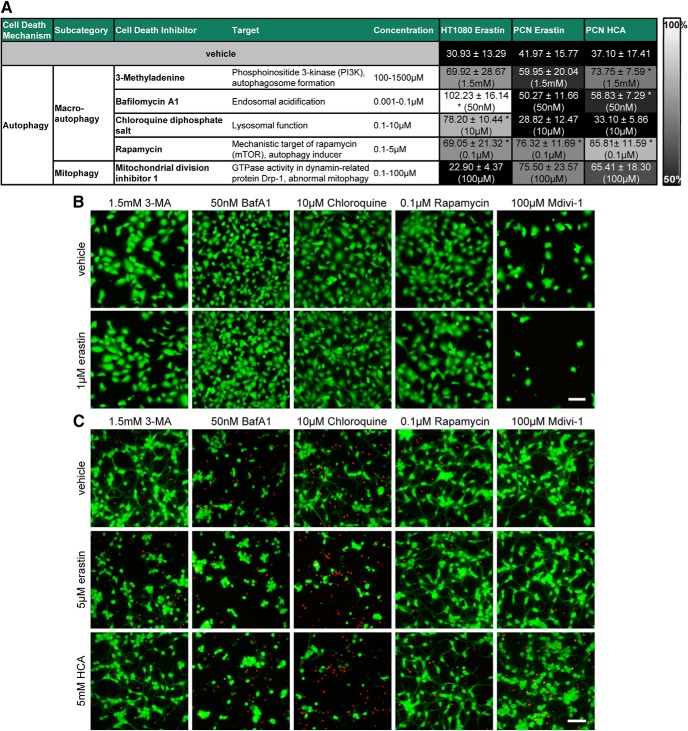
Autophagy inhibitors inhibit ferroptosis in cancer cells (HT1080) and primary cortical neurons (PCNs). ***A***, HT1080 cells were treated with 1 µM erastin, PCN with 5 µM erastin or 5 mM glutamate analog HCA and chemical inhibitors effective in autophagy were examined. Numbers show mean ± SD at representative concentration in brackets. Grayscale coding indicates the continuum from no protection in the presence of erastin (black) to maximal cell viability (white). *p < 0.05 versus erastin or glutamate analog (HCA). ***B***, ***C***, Representative live/dead staining in HT1080 cells (***B***) and PCN (***C***) are shown, green indicating live cells (calcein AM) and red indicating dead cells (ethidium homodimer-1). Scale bar, 50 µm.

**Figure 9.**
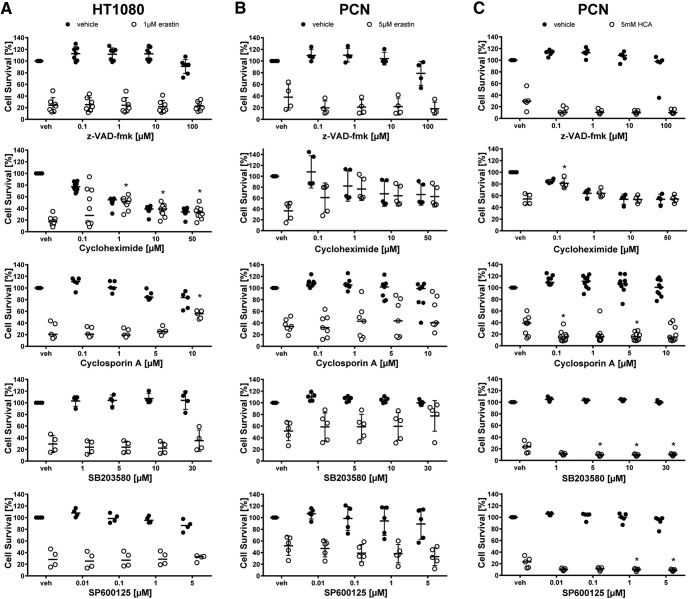
Dose–responses of autophagy inhibitors in cancer cells (HT1080) and primary cortical neurons (PCNs). HT1080 cells were treated with 1 µM erastin, PCNs with 5 µM erastin or 5 mM glutamate analog HCA and chemical inhibitors effective in autophagy were examined. Values represent mean ± SD, except for 3-methyladenine, Bafilomycin A1, and Chloroquine in PCNs treated with erastin as well as all inhibitors in PCNs treated with glutamate analog (HCA) where medians are given. *p < 0.05 versus erastin or glutamate analog (HCA). For exact *p* values refer to Figure 9-1.

Unexpectedly, several modulators of autophagy (Bafilomycin A1, Chloroquine, and rapamycin; (Pasquier, 2016) abrogated erastin-induced cell death in HT1080 fibrosarcoma cells. In primary neurons, the autophagy inducer rapamycin decreased both erastin- and glutamate analog (HCA)-induced toxicity. In addition, we found a significant increase in viability with the autophagy inhibitors 3-methyladenine and Bafilomycin A1 in glutamate analog (HCA)-induced neuronal death (Figs. 8, 9).

Necroptosis is another mode of regulated cell death that depends on the activation of receptor-interacting proteins (RIPs; Degterev et al., 2005; Sun et al., 2012). The RIP1 inhibitor Necrostatin-1 abrogated erastin-induced cell death in HT1080 cells and primary neurons as well as glutamate analog (HCA)-induced neuronal toxicity. In addition, the RIP3 inhibitor GSK872 blocked neuronal cell death from erastin and glutamate analog (HCA) and the inhibitor of mixed lineage kinase domain-like protein (MLKL), necrosulfonamide, prevented erastin toxicity in HT1080 cells (Figs. 6, 7).

**Figure 10.**
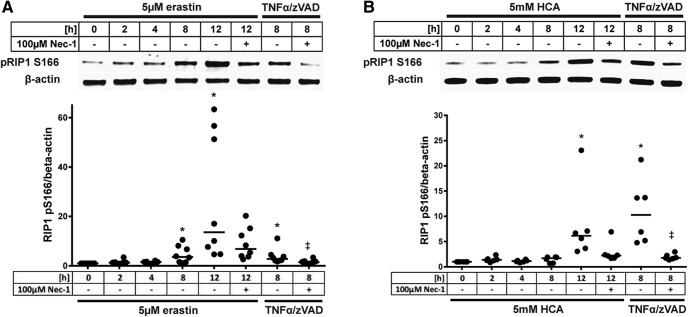
Ferroptosis inducer erastin and glutamate analog HCA activate necroptotic machinery. Levels of phospho-RIP1 (normalized to β-actin) were measured in HT22 cells exposed to erastin (***A***), glutamate analog (HCA; ***B***) or 100 ng/ml TNFα + 5 µM z-VAD-fmk for 8 h (positive control in ***A*** and ***B***). Necrostatin-1 served as confirmation for specificity of RIP1 kinase activity. Values represent median. *p < 0.05 versus 0 h erastin or glutamate analog (HCA), ‡*p* < 0.05 versus 8 h TNFα/zVAD. For exact *p* values refer to Figure 10-1.

The specificity of Necrostatin-1 against RIP1 was suggested by a structural analog (Necrostatin-1i), with no activity toward RIP1, that did not block erastin- or glutamate analog (HCA)-induced toxicity. Moreover, as RIP1 kinase activity is required to execute necroptosis (Berger et al., 2014), we assessed the ability of erastin to induce active RIP kinase using an antibody against a known RIP1 autophosphorylation site at serine 166 (Guo et al., 2015). We found a Necrostatin-1-sensitive increase in phospho-RIP1 following erastin and glutamate analog (HCA) treatment in HT22 mouse hippocampal neuronal cells (Fig. 10). These cells also died in response to both inducers of cyst(e)ine/glutathione depletion.

**Figure 11.**
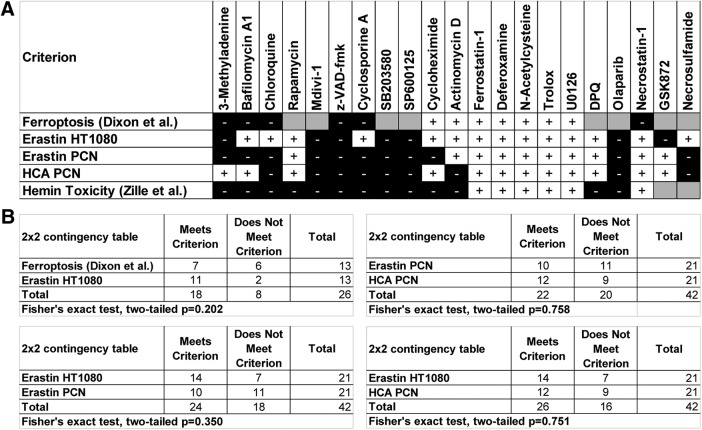
Systematic pharmacologic characterization reveals inhibitors of autophagy and necroptosis abrogate ferroptosis in cancer cells (HT1080) and primary cortical neurons (PCNs). ***A***, Comparison of protection profile of chemical inhibitors between operationally defined ferroptosis (Dixon et al., 2012, 2014), erastin-induced toxicity in HT1080 cells at toxicologically meaningful dose, erastin and glutamate analog (HCA) in PCNs and previously published hemin toxicity in PCNs (Zille et al., 2017). ***B***, Statistical analysis of profile of chemical inhibitors between operationally defined ferroptosis and erastin-induced toxicity in HT1080 revealed that they are statistically similar, but major differences (i.e., protection by inhibitors of autophagy and necroptosis) exist. Statistical analysis showed no difference between erastin treatment in PCNs and HT1080 cells, erastin- and glutamate analog (HCA)-treated PCNs or erastin in HT1080 cells and HCA in PCNs.

To elucidate whether erastin- and glutamate analog (HCA)-induced toxicities are similar or distinct modes of cell death (Fig. 11*A*), we performed statistical comparisons between the inhibitor profiles (Fig. 11*B*). First, we found that erastin toxicity in HT1080 fibrosarcoma cells can be considered ferroptosis as previously described (Dixon et al., 2012; Fisher’s exact test, two-tailed, *p* = 0.202), despite the ability of the inducers of autophagy or the inhibitors of necroptosis to prevent “ferroptosis” at the lower dose (1 µM) in HT1080 cells. Second, erastin toxicity in HT1080 fibrosarcoma cells and in neurons can be considered mechanistically similar (Fisher’s exact test, two-tailed, *p* = 0.350). Similarly, erastin and glutamate analog (HCA) toxicity in neurons can be considered mechanistically similar (Fisher’s exact test, two-tailed, *p* = 0.758).

Collectively, these findings suggest that erastin induces cell death in both cancer cells and neurons through ferroptotic, autophagic, and necroptotic pathways and that they are not different from what is known from the established glutathione depletion model referred to incorrectly as apoptosis (Ratan et al., 1994b) or correctly as oxytosis (Lewerenz et al., 2018) in prior studies (Fig. 11*B*; Fisher’s exact test, two-tailed, *p* = 0.751). Convergence of these distinct modes of cell death in a single cell death paradigm has not been described so far.

### Promoting cancer cell death while protecting neurons

**Figure 12.**
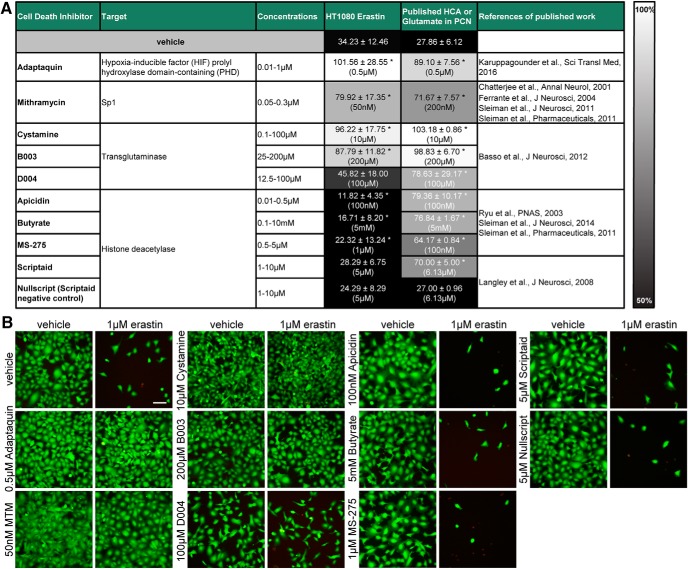
Do inhibitors of HCA-induced death in neurons prevent ferroptosis in cancer cells? ***A***, HT1080 cells were treated with 1 µM erastin (LD_50_) and chemical inhibitors effective in glutamate analog (HCA)-induced neuronal toxicity (values from published work and references indicated in last 2 columns) were examined. Numbers show mean ± SD at representative concentration in brackets. Grayscale coding indicates the continuum from no protection in the presence of erastin (black) to maximal cell viability (white). *p < 0.05 versus erastin, #*p* < 0.05 versus Nullscript (negative control). ***B***, Representative live/dead staining are shown, green indicating live cells (calcein AM) and red indicating dead cells (ethidium homodimer-1). Scale bar, 50 µm.

**Figure 13.**
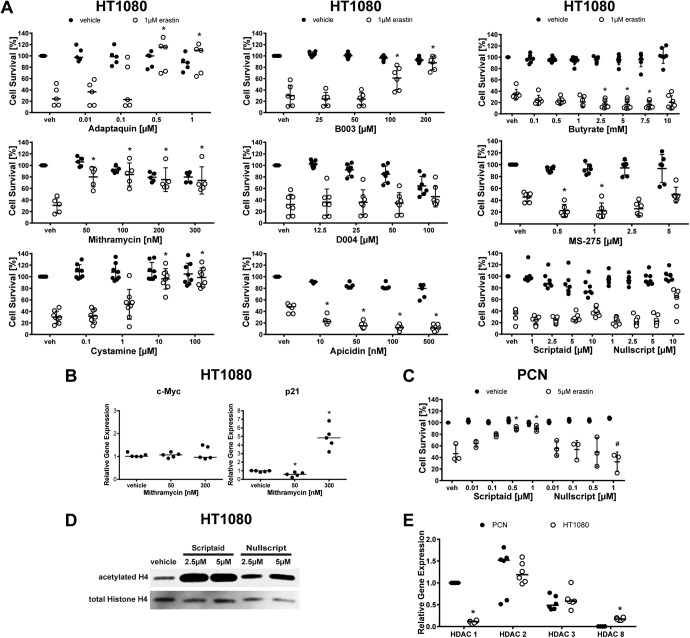
Protective effect of inhibitors of neuronal toxicity of glutamate analog (HCA) in erastin-induced death in HT1080 cells. ***A***, Dose–responses of chemical inhibitors effective in HCA-induced neuronal toxicity. Values represent mean ± SD, except for Adaptaquin, Apicidin, and Scriptaid, where medians are given. *p < 0.05 versus erastin. ***B***, Gene expression levels of Mithramycin target genes c-Myc and p21 were assessed in HT1080 cells exposed to 50 or 300 nm Mithramycin. Values represent medians. *p = 0.009 versus vehicle. ***C***, Dose–responses of Scriptaid and Nullscript (negative control) in erastin-induced cell death in primary cortical neurons (PCNs). Values represent mean ± SD, *p < 0.05 versus erastin, #*p* < 0.05 versus Nullscript (negative control). ***D***, Protein levels of acetylated and total histone H4 were assessed in HT1080 cells exposed to Scriptaid or its inactive analog Nullscript. ***E***, Gene expression of HDACs from class I in PCNs versus HT1080 cells. Values represent medians. *p < 0.0125 versus PCNs. For exact *p* values refer to Figures 13-1, 13-2, 13-3, and 13-4.

Because erastin toxicity in HT1080 cells and glutamate analog (HCA)-induced glutathione depletion in neurons induce ferroptotic cell death, we hypothesized that prior knowledge about glutamate analog (HCA)-induced ferroptosis in neurons should inform the points of convergence and divergence with erastin-induced ferroptosis in HT1080 fibrosarcoma cells. We therefore investigated whether erastin-induced ferroptosis in HT1080 can be abolished or exacerbated by the chemical inhibitors known to be effective in glutamate analog (HCA)-induced neuronal ferroptosis (Figs. 12, 13*A*).

Glutamate analog (HCA)-induced ferroptosis in neurons requires *de novo* transcription via the leucine zipper transcription factor ATF4 leading to upregulation of putative pro-death genes such as *Trib3*, *Chop*, and *Chac1* (Lange et al., 2008; Karuppagounder et al., 2016). Recently, it was shown that HIF prolyl hydroxylases (HIF PHDs) are required for pro-death ATF4 transcription, and a selective small molecule inhibitor of the HIF PHDs, Adaptaquin, abrogates glutamate analog (HCA)-induced ferroptosis and improves functional recovery after intracerebral hemorrhage, where cell death has also been defined as ferroptotic (Karuppagounder et al., 2016). As expected from these findings, Adaptaquin also protected against erastin-induced toxicity in HT1080 cells (Figs. 12, 13*A*).

Prior studies have shown that, like Adaptaquin, the aureolic acid antitumor agent Mithramycin can also act in the nucleus to abrogate glutamate analog (HCA)-induced ferroptosis *in vitro* and extend survival *in vivo* in an HD model, where ferroptosis has also been implicated (Chatterjee et al., 2001; Ferrante et al., 2004; Sleiman et al., 2011a, b). Mithramycin inhibits ferroptosis by acting in a gene-selective way to inhibit Sp1-dependent genes. For example, oncogenic and pro-death c-Myc is suppressed by Mithramycin, but protective p21 waf1/cip1 is induced. Given the ability of this established anticancer drug to abrogate glutamate analog-induced ferroptosis in neurons, we examined its effect in erastin-induced cell death in HT1080 cells. Unexpectedly, Mithramycin (50–300 nm) abrogated erastin-induced death in cancer cells (Figs. 12, 13*A*). We confirmed that Mithramycin was able to induce p21 gene expression in HT1080 cells, but failed to reduce basal c-Myc levels suggesting the possibility that augmentation of tumor suppression genes rather than suppression of proto-oncogenes may mediate Mithramycin’s effects in HT1080 cells (Fig. 13*B*; Sleiman et al., 2011b).

Transglutaminases are established transcriptional targets for Huntington’s disease (McConoughey et al., 2010) and have been shown to mediate glutamate analog (HCA)-induced ferroptosis via their effects in the nucleus, downstream of the hyperactivation of ERK signaling (a cardinal feature of ferroptosis; Basso et al., 2012). As transglutaminases have also been implicated as survival factors for tumors, we reasoned that the inhibitors of transglutaminases may protect neurons while exacerbating HT1080 fibrosarcoma-induced ferroptosis. However, two structurally diverse transglutaminase inhibitors also inhibited erastin-induced ferroptosis in HT1080 fibrosarcoma cells (Figs. 12, 13*A*), further demonstrating convergence between cyst(e)ine deprivation in transformed fibrosarcoma cells and non-transformed neurons.

Another class of agents with known antitumor activity shown to abrogate HCA-induced ferroptosis are class I HDAC inhibitors (Ryu et al., 2003; Langley et al., 2008; Sleiman et al., 2011a, 2014). Structurally diverse HDAC inhibitors including Butyrate, Scriptaid, and Trichostatin A, all prevent HDAC-induced ferroptosis in neurons (Ryu et al., 2003). By contrast to Adaptaquin, Mithramycin A, and transglutaminase inhibitors, class I HDAC inhibitors failed to protect HT1080 cells from erastin toxicity, in fact, they exacerbated cell death (Figs. 12, Fig. 13*A*). In contrast, Scriptaid, which was able to protect neurons from ferroptosis (Fig. 13*C*), did not exacerbate ferroptosis in cancer cells. We confirmed that Scriptaid but not its structurally similar, inactive analog Nullscript, increased the acetylation of histones using Western blotting (Fig. 13*D*).

We then hypothesized that the expression of distinct HDAC isoforms in neurons versus cancer cells explains this difference. We therefore assessed the gene expression of HDACs from class I and found that HT1080 cells and primary neurons express HDAC1, 2, and 3. Surprisingly, only HT1080 cells express HDAC8, raising the intriguing possibility that HDAC 8 expression may explain the differential effects of HDAC inhibitors between HT1080 cells and primary neurons (Fig. 13*E*).

**Figure 14.**
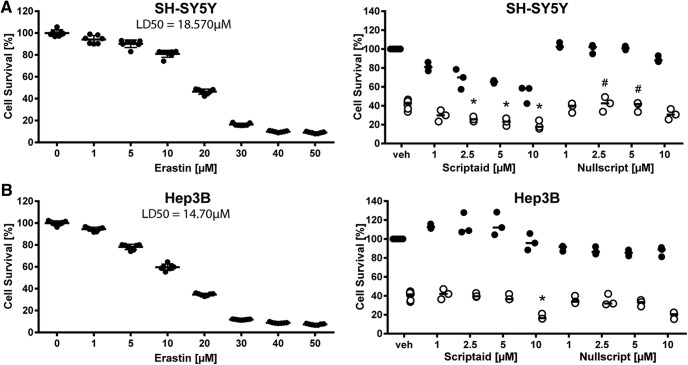
Dose–responses of erastin and Scriptaid in SH-SY5Y neuroblastoma and Hep3B hepatocellular carcinoma cells. SH-SY5Y (***A***) and Hep3B cells (***B***) were treated with increasing dose of erastin to determine the LD_50_ that was subsequently used to assess whether Scriptaid exacerbated erastin-induced toxicity. Nullscript was used as structural analog (negative control). Values represent mean ± SD. *p < 0.05 versus erastin, #*p* < 0.05 versus Nullscript. For exact *p* values refer to Figure 14-1.

To determine whether the effect is specific to HT1080 cells or applicable to other cancer cells, we evaluated whether Scriptaid protected against erastin-induced toxicity in the neuroblastoma cell line SH-SY5Y and the hepatocellular carcinoma cell line Hep3B (at the LD_50_ of erastin for each cell line). We found that 2.5-10 µm Scriptaid exacerbated erastin-induced cell death in SH-SY5Y cells, whereas the negative control did not. However, it also decreased viability of SH-SY5Y cells without erastin treatment (Fig. 14*A*). In contrast, 10 µM Scriptaid also enhanced erastin toxicity in Hep3B cells, although it was not toxic for the cells at that dose (Fig. 14*B*). Altogether, these findings suggest that HDAC inhibitors could be good adjunctive treatment for erastin or erastin-like drugs in fibrosarcomas, neuroblastoma, and hepatomas.

## Discussion

The objective of this study was to understand the mechanisms by which erastin (an X_c_^−^ transporting agency inhibitor) induces death in cultured CNS neurons compared with erastin-induced death in cancer cells. The X_c_^−^ transporting agency is a chloride-dependent transporter that exchanges cystine for glutamate. Cystine is converted intracellularly to cysteine, which is used for protein synthesis and synthesis of the versatile antioxidant glutathione. Depletion of cyst(e)ine leads to depletion of glutathione and reactive lipid species-induced death now defined as ferroptosis.

Our ultimate goal in pursuing these studies is to develop combinatorial therapeutic approaches that on the one hand enhance erastin-induced death of tumor cells, and on the other, prevent its potential toxicities in the nervous system mediated via erastin-induced neuronal death. Our results suggest that cell death pathways induced by erastin in post-mitotic cortical neurons and HT1080 fibrosarcoma are similar to each other and to oxidative glutamate toxicity in neurons (Figs. 2-9, 11). Both glutamate and erastin can inhibit the system X_c_^−^ to induce death in immature neurons where functional, ionotropic glutamate receptors are not expressed (Murphy et al., 1989; Ratan et al., 1994a, b,). We leveraged prior studies of cystine deprivation in neurons to identify HDAC inhibitors as agents that enhance the chemotherapy-induced ferroptosis of tumors while inhibiting ferroptosis in neurons. These findings suggest a combinatorial approach to cancer chemotherapy (erastin plus a class I HDAC inhibitor) designed to maximize tumor cell death and minimize neuronal toxicity. Our studies also identified agents that prevent ferroptotic death in cancer cells and neurons that would not be good candidates for cancer treatment (e.g., HIF PHD inhibitors, transglutaminase inhibitors, and the gene selective Sp1 inhibitor Mithramycin).

Our studies systemically evaluated whether erastin-induced ferroptosis in cancer cells occurs via mechanisms that are similar to or distinct from those induced by erastin in primary neurons. We found that erastin-induced toxicity in tumor cells can be abolished not only by previously published ferroptosis inhibitors (Dixon et al., 2012; Figs. 2, 3) but also by inhibitors of necroptosis (Figs. 6–7, 10) and autophagy (Figs. 8, 9) when erastin was used at a toxicologically relevant dose.

With respect to autophagy, Torii et al. (2016) previously reported that Bafilomycin A1, a vacuolar ATPase inhibitor that prevents endosomal acidification, abolished cell death induced by erastin (all doses tested) as well as another inducer of ferroptosis, RSL3, in HT1080 and Calu-1 cells. Similarly, knock-out of autophagy-related genes Atg5 and Atg7 in mouse embryonic fibroblasts and knockdown in HT1080 cells partially inhibited erastin-induced cell death (Hou et al., 2016). These data suggest that cyst(e)ine deprivation may not only be interpreted by the cell as uncompensated redox but also nutrient dyshomeostasis, thereby triggering multiple parallel pathways (e.g., reactive lipid species, autophagy) that collectively elevate the threshold of the cell toward cell demise (Zille et al., 2017).

With respect to necroptosis, Yu and colleagues demonstrated that Necrostatin-1 as well as knockdown of RIP3 abrogated erastin toxicity in acute myeloid leukemia cells (Yu et al., 2015). Interestingly, MLKL deficiency increased sensitivity to ferroptosis stimuli in mouse embryonic fibroblast. In turn, the loss of acyl-CoA synthetase long-chain family member 4, that suppresses ferroptosis by limiting the membrane-resident pool of oxidation-sensitive fatty acids, predisposed cells to necroptosis (Muller et al., 2017). This suggests that necroptosis and ferroptosis are differentially regulated cell death pathways. It has previously been demonstrated that ferroptosis and necroptosis are recruited independently in neurons exposed to intracerebral hemorrhage and that they may then converge at a yet to be identified common denominator leading to a necrotic morphology (Zille et al., 2017). Here, we provide evidence that erastin is able to recruit the necroptotic machinery in diverse cell types (Fig. 10).

However, HT1080 fibrosarcoma cells have been reported not to express RIP3 (de Almagro et al., 2017) and were therefore not responsive to the inhibition by GSK872 in our study. This is interesting, because Necrostatin-1 and necrosulfonamide abolished erastin-induced toxicity. Although the activation of RIP1, RIP3, and MLKL have been considered to be indispensable for necroptosis, recent studies suggest possible necroptosis-independent roles of each protein, including the induction of apoptosis and the inflammasome (Mandal et al., 2014; Berger et al., 2016). In addition to the mentioned differences in the expression and post-translational regulation of necrosome complex proteins and caspase-8 in various cell types (Newton et al., 2016; de Almagro et al., 2017), they also differ in their sensitivity to erastin (Yang et al., 2014; Yu et al., 2015). Thus, the relative contribution of ferroptosis and necroptosis during regulated cell death needs further investigation, especially with respect to cell type, tissue, and disease. This will greatly influence the effectiveness and toxicity of chosen chemotherapeutic approaches.

To investigate the ability of erastin to cause unwanted toxicities in the nervous system, we examined whether erastin engages a similar pattern of cell death signaling in primary neurons compared with HT1080 cells. Indeed, the exposure to erastin led to the death of primary cortical neurons, which was reversed by a statistically similar profile (Fig. 11) of inhibitors of cell death, including autophagy (Figs. 8, 9), ferroptosis (Figs. 2, 3), and necroptosis (Figs. 6, 7, 10). We also found that in an established glutathione depletion model involving non-receptor-mediated toxicity by glutamate (HCA), a panoply of chemical inhibitors also blocked erastin-induced death (Figs. 2-9, 11). In addition, both erastin- and glutamate (HCA)-induced ferroptosis recruited the necrosome by activating RIP1 (Fig. 10). This is further substantiated by recent findings of Neitemeier et al. (2017) who demonstrated mitochondrial dysfunction in glutamate and erastin toxicity in HT22 hippocampal neuroblast cells, and this effect was abolished by the loss of BH3 Interacting Domain Death Agonist (BID).

Finally, we sought to demonstrate whether the knowledge about glutamate analog (HCA)-induced ferroptosis/oxytosis in neurons can be applied to ferroptosis in cancer cells (Figs. 12, 13). Indeed, we found that Adaptaquin, transglutaminase inhibitors, and Mithramycin all protect cancer cells from erastin similar to their protection in neuronal toxicity. However, HDAC inhibitors exacerbated erastin toxicity in cancer cells while promoting survival in neurons. Collectively, our data indicate that a combination of anticancer drugs may reverse the effect of single agents (as it is the case for erastin and Mithramycin), and that selectively targeting cancer cells while sparing neurons may be a more promising therapeutic approach.

It is important to note that sulfasalazine, which functions as an X_c_^−^ transport inhibitor like erastin, has been shown to not only inhibit glial-derived tumor (glioma) growth but also to inhibit neuronal excitotoxic death and seizures associated with these tumors (Chung et al., 2005; Robert et al., 2015). Under these circumstances, sulfasalazine confers salutary effects on neurons by preventing the release of glutamate from gliomas. Indeed, glioma growth depends on glutamate release, and this may be a mechanism by which tumor cells remove neurons that compete for space and nutrients. These results suggest that erastin may actually prevent some toxicities to the CNS that are derived from tumors, rather than creating toxicities on its own.

However, several observations potentially reconcile our results with seminal studies from Sontheimer et al. First, they evaluated the effects of X_c_^−^ transport inhibition with only one drug concentration (sulfasalazine) where there is clear sensitivity of the glioma tumor to glutathione depletion but no effect on neurons or astrocytes. Indeed, studies here clearly demonstrate that cortical neurons are sensitive to X_c_^−^ transport inhibition with an LD_50_ 5-fold higher than those required to kill 50% of the HT1080 cells. Second, they did not evaluate toxicity to peripheral neurons or central neurons directly, still leaving the question open whether X_c_^−^ inhibition systemically can lead to chemotherapy-induced neuropathy or chemo brain. Accordingly, apparent toxicity may occur under circumstances where drug concentrations used to kill tumor cells are higher and begin to approach the susceptibility of neurons. HDAC inhibitors in combination with erastin appear to be a viable strategy to minimize toxicity as they would reduce the concentration of erastin or sulfasalazine required for killing but would provide for neuroprotection from ferroptosis in their own right.

In conclusion, cell death pathways in cancer cells and neurons exposed to glutathione depletion are similar as judged by the Fisher’s tests. Our data suggest that HDAC inhibitors are promising therapeutic agents for combinatorial cancer chemotherapy to enhance chemotherapy-induced ferroptosis of tumors while inhibiting ferroptosis in neurons. The current study further identifies agents, some of which are known antitumor drugs, which would prevent erastin-induced toxicity in neurons (e.g., Adaptaquin, Mithramycin, transglutaminase inhibitors) and in cancer cells suggesting that these are not good combinatorial therapies to optimally kill tumor cells while preserving CNS and PNS neurons.

## Synthesis

Reviewing Editor: Julie Andersen, Buck Institute Decisions are customarily a result of the Reviewing Editor and the peer reviewers coming together and discussing their recommendations until a consensus is reached. When revisions are invited, a fact-based synthesis statement explaining their decision and outlining what is needed to prepare a revision will be listed below.

The reviewers and myself felt that the scientific premise behind the study was high potential therapeutic significance--identification of inhibitors specific for cancer cell death which avoid neuronal toxicity and, in particular, the identification of ferroptosis inhibition as a mechanistic target and HDAC inhibitors as a potential class of drugs. for this purpose. It was felt by all that the manuscript would however benefit from some re-organization and additional background to make it more accessible to readers unfamiliar with the subject area, such as inclusion of a graphical summary. The issue of lack of any confirmational in vivo data was also raised, which should be addressed. There may be a need for additional in vitro experiments to address the eviewers' issues or questions.

Major Concerns:

1) The most compelling part of the study is the potential to identify inhibitors that will protect neurons but not protect cancer cells. Although the authors have done a side by side comparison of HT1080 cells and primary cortical neurons for essentially all the experiments, they then switch to analysis of the published literature for neurons and provide their own analysis of HT1080 cells. These are all inhibitors that were identified when looking at glutamate analog-induced ferroptosis in neurons, so it will be critical to demonstrate that the protective effect for PCNs in glutamate analog-induced death also occurs during erastin treatment. Early in the manuscript, the authors highlight the importance of carefully identifying appropriate doses and cell confluence to use in order to clearly see treatment effects. To follow this principle, they should carry out these experiments testing Erastin treatment and this set of inhibitors on PCNs in their own hands.

2) While the organization and logic of individual figures is relatively evident, the organization of the text in the results is a bit difficult to follow. I would suggest an alternative organization (which might require reworking of some figures)

• Explain how ferroptosis works (cysteine deprivation) and how it is operationally defined by sensitivity to various inhibitors → then present data demonstrating that erastin is working in this way in both HT1080 cells and PCNs

• Explain how glutamate and its analogs induce cell death via inhibition of system Xc, a mechanism that was elucidated in 1980's and has convergence with erastin induced death which also operates via cysteine deprivation → then present data that glutamate analog death in PCNs and erastin death in PCNs both shows the same pattern of sensitivity to ferroptosis inhibitors

• Then explain that there are many other modes of cell death (apoptosis, parthanosis - with additional, brief descriptions of how these modes of cell death operate) → and present data showing that HT1080 erastin death, PCN erastin death, and PCN HCA death is not altered by inhibitors of these types of cell death

• Then show evidence that, surprisingly, inhibitors of autophagy and necroptosis also blunted erastin cell death. In the results text or the discussion, consider how this discrepancy with results from previous studies could be because they used LD95 and difference cell confluence, instead of carefully identifying LD50 to use

- also here can include the data about phospho-RIP1 as evidence that erastin really is engaging necroptosis machinery (currenty figure 10)

• Now, given this more complete picture of the pattern of erastin induced cell death and sensitivity to different types of inhibitors, can we find something that will protect neurons but not protect cancer cells? Present data about final group of HCA-death inhibitors including their ability (or lack of ability) to protect HT1080 cells and PCNs from Erastin induced death

3) While the authors have been very comprehensive in their approach, the study is carried out exclusively in cultured cells. Many cell types behave very differently in vitro than they do in vivo. In addition, primary cortical neurons are derived from fetal tissue and may show different patterns of sensitivity and resilience compared to adult neurons. The manuscript would be greatly enhanced by any minimal indications of whether this will be relevant in vivo. The ideal scenario would be to treat mice with Erastin and HDAC inhibitors and evaluate neuronal cell death. Huo and Gu 2016 PLOS ONE treat mice with i.p. injections of Erastin. Or treat scid mice with engrafted tumors with Erastin and HDAC inhibitors to show that Erastin is still effective in limiting tumor growth.

If the authors are not equipped to do these types of studies with adult mice, some additional in vitro experiments that could expand the significance of the study would be:

- Profiling expression levels of gpx4 in different cancer cell lines and neuronal lines or different primary neuron types to better speculate about relative sensitivity to erastin treatment death

- Profiling expression levels of different hdacs in cancer cells and neuronal cell lines or different primary neuron types to better speculate about utility of this protective strategy

- test several other relevant cancer cell lines to show whether the HDAC inhibitors can protect PCNs and still permit erastin death of those cancer cell lines.

Additional concerns:

1) Figure 1 shows how the toxicologically meaningful dose of Erastin (1uM) was established for HT1080 cells. Presumably, this same sort of dose-response analysis was also carried out to determine what dose of Erastin to use for the PCNs (5 uM) - this data should be shown.

2) For readers who are not in the field, the authors should provide more background information:

a. What are the characteristics of HT10180 cells - why were they chosen for the study?

b. Define MTT assays.

c. Include a figure that presents a diagram of the different types of cell death pathways and where the pharmacological agents used in the study are acting

d. Why are cancer cells thought to be more sensitive to this form of cell death? Higher expression of system XC? Do cancer cells need more antioxidant protection in general?

3) The authors could simply state the IC50's for the different cells found in Figure 1 in the body of the text, rather than display the whole figure.

Figure 2 should show the vehicle alone pictures.

4) In Figures 2-5 I am not clear what the numbers in the table represent. Is there a better way to display them?

5) Are statistics based on each well in single experiments? If so, have the experiments been repeated?

6) Figure 2: Actinomycin and UO126 images in PCN do not match survival numbers.

7) Figure 3: Baflomycin and Chloroquine images in PCN do not match survival numbers.

8) Figures 6-8 should be combined into 1 figure highlighting the inhibitors that have a differential effect on normal vs. cancer cells.

9) Figure 2: “U0126”; is written in the table and “U0124” in the figure.

## Author Response

We would like to thank the reviewers for the helpful and instructive comments of our manuscript entitled, “Ferroptosis in cancer cells and primary neurons is similar but is differentially regulated by HDAC inhibitors”. We have revised the manuscript according to the reviewer's suggestions. We believe that this significantly improved the clarity of our results and the interpretation of the data.

Manuscript Instructions

- Extended Data must be named after the figure or table in the main manuscript that it supports. For example, any Extended Data supporting Figure 2, whether the Extended Data is a figure, table, or Excel file, should be labeled as Figure 2-1, Figure 2-2, etc. Similarly any Extended Data supporting Table 2 would be labeled as Table 2-1, Table 2-2, etc. Extended Data should be labeled as Figure 1-1, Figure 1-2, Table 1-1, etc., so they indicate which figure they are supporting (i.e. Extended Data table supporting Figure 2 labeled as Figure 2-1).

Response: We have changed this according to the rules of the journal.

Synthesis Statement for Author (Required):

The reviewers and myself felt that the scientific premise behind the study was high potential therapeutic significance--identification of inhibitors specific for cancer cell death which avoid neuronal toxicity and, in particular, the identification of ferroptosis inhibition as a mechanistic target and HDAC inhibitors as a potential class of drugs. for this purpose. It was felt by all that the manuscript would however benefit from some re-organization and additional background to make it more accessible to readers unfamiliar with the subject area, such as inclusion of a graphical summary. The issue of lack of any confirmational in vivo data was also raised, which should be addressed. There may be a need for additional in vitro experiments to address the reviewers' issues or questions.

Response: We have added a graphical abstract according to thoughtful suggestion of the editors.

Major Concerns:

1) The most compelling part of the study is the potential to identify inhibitors that will protect neurons but not protect cancer cells. Although the authors have done a side by side comparison of HT1080 cells and primary cortical neurons for essentially all the experiments, they then switch to analysis of the published literature for neurons and provide their own analysis of HT1080 cells. These are all inhibitors that were identified when looking at glutamate analog-induced ferroptosis in neurons, so it will be critical to demonstrate that the protective effect for PCNs in glutamate analog-induced death also occurs during erastin treatment. Early in the manuscript, the authors highlight the importance of carefully identifying appropriate doses and cell confluence to use in order to clearly see treatment effects. To follow this principle, they should carry out these experiments testing Erastin treatment and this set of inhibitors on PCNs in their own hands.

Response: Our data indicates that glutamate-analog- and erastin-induced cell death in PCNs are essentially identical in that they are both inhibited by macromolecular synthesis inhibitors, inhibitors of reactive lipids, iron chelators, and ERK inhibitors (Fig. 9). We have added the values of published work on the different inhibitors in HCA/glutamate-induced toxicity in PCNs (Fig. 12). In addition, we have also included our own data on Scriptaid in erastin-induced cell death in PCNs (Figure 13C).

2) While the organization and logic of individual figures is relatively evident, the organization of the text in the results is a bit difficult to follow. I would suggest an alternative organization (which might require reworking of some figures)

• Explain how ferroptosis works (cysteine deprivation) and how it is operationally defined by sensitivity to various inhibitors → then present data demonstrating that erastin is working in this way in both HT1080 cells and PCNs

• Explain how glutamate and its analogs induce cell death via inhibition of system Xc, a mechanism that was elucidated in 1980's and has convergence with erastin induced death which also operates via cysteine deprivation → then present data that glutamate analog death in PCNs and erastin death in PCNs both shows the same pattern of sensitivity to ferroptosis inhibitors

• Then explain that there are many other modes of cell death (apoptosis, parthanosis - with additional, brief descriptions of how these modes of cell death operate) → and present data showing that HT1080 erastin death, PCN erastin death, and PCN HCA death is not altered by inhibitors of these types of cell death

• Then show evidence that, surprisingly, inhibitors of autophagy and necroptosis also blunted erastin cell death. In the results text or the discussion, consider how this discrepancy with results from previous studies could be because they used LD95 and difference cell confluence, instead of carefully identifying LD50 to use

- also here can include the data about phospho-RIP1 as evidence that erastin really is engaging necroptosis machinery (currenty figure 10)

• Now, given this more complete picture of the pattern of erastin induced cell death and sensitivity to different types of inhibitors, can we find something that will protect neurons but not protect cancer cells? Present data about final group of HCA-death inhibitors including their ability (or lack of ability) to protect HT1080 cells and PCNs from Erastin induced death

Response: We agree with the reviewer and have reorganized the results section accordingly.

3) While the authors have been very comprehensive in their approach, the study is carried out exclusively in cultured cells. Many cell types behave very differently in vitro than they do in vivo. In addition, primary cortical neurons are derived from fetal tissue and may show different patterns of sensitivity and resilience compared to adult neurons. The manuscript would be greatly enhanced by any minimal indications of whether this will be relevant in vivo. The ideal scenario would be to treat mice with Erastin and HDAC inhibitors and evaluate neuronal cell death. Huo and Gu 2016 PLOS ONE treat mice with i.p. injections of Erastin. Or treat scid mice with engrafted tumors with Erastin and HDAC inhibitors to show that Erastin is still effective in limiting tumor growth.

If the authors are not equipped to do these types of studies with adult mice, some additional in vitro experiments that could expand the significance of the study would be:

- Profiling expression levels of gpx4 in different cancer cell lines and neuronal lines or different primary neuron types to better speculate about relative sensitivity to erastin treatment death

- Profiling expression levels of different hdacs in cancer cells and neuronal cell lines or different primary neuron types to better speculate about utility of this protective strategy

- test several other relevant cancer cell lines to show whether the HDAC inhibitors can protect PCNs and still permit erastin death of those cancer cell lines.

Response: We agree that the in vivo experiments are beyond the scope of the current manuscript. We have added the following in vitro data to the manuscript according to some of the suggestions of the reviewer:

Erastin dose-response in dorsal root ganglia sensory neurons (Fig. 1D). These data suggest that erastin could not only cause chemo-brain but also chemotherapy induced neuropathy.

Gene expression of class I HDAC inhibitors in PCN and HT1080 cells (Fig. 13E). These data suggest that the differential sensitivity of HT1080 fibrosarcoma cells and primary neurons to class I HDAC inhibitors may depend on differential expression of HDAC8.

Erastin and scriptaid dose-responses in the neuroblastoma cell line SH-SY5Y and hepatocellular carcinoma cell line Hep 3B (Fig. 14). Accordingly, other tumor cells behave similarly to HT1080 in not being protected from ferroptosis by HDAC inhibitors.

Additional concerns:

1) Figure 1 shows how the toxicologically meaningful dose of Erastin (1uM) was established for HT1080 cells. Presumably, this same sort of dose-response analysis was also carried out to determine what dose of Erastin to use for the PCNs (5 uM) - this data should be shown.

Response: We have provided erastin and HCA (glutamate analog) dose-responses in PCNs in Figure 1.

2) For readers who are not in the field, the authors should provide more background information:

a. What are the characteristics of HT10180 cells - why were they chosen for the study?

Response: The HT1080 cells were used in the original study on ferroptosis by Dixon et al. contain an activating mutation of NRAS. 30% of all tumors have RAS mutations which makes them vulnerable to ferroptotic death induced by Xc- transport inhibition by depletion of glutathione. We have now included other cancer cells in this study, see also our response to major concern number 3.

b. Define MTT assays.

Response: MTT is 3-(4,5-dimethylthiazol-2-yl)-2,5-diphenyltetrazolium bromide and this is a colorimetric assay of cell metabolic activity. Prior studies from our lab have shown that MTT is converted to blue formazan by mitochondrial or glycolytic reductases. In cortical neurons, there is a linear relationship between blue formazan (which can be solubilized by detergent) and measured spectrophotometrically and cell number. We have added this in the methods section.

c. Include a figure that presents a diagram of the different types of cell death pathways and where the pharmacological agents used in the study are acting

Response: We have included this in Figure 1 for ferroptosis.

d. Why are cancer cells thought to be more sensitive to this form of cell death? Higher expression of system XC? Do cancer cells need more antioxidant protection in general?

Response: This is a great question, and we do not have a clear answer for at this point. ERK activation via redox inhibition of serine/threonine phosphatases is thought to be a mechanism of cell death in primary neurons (Stanciu et al., 200). Hyperactive ERK is believed to translocate to the nucleus where it mediates death, although its precise targets are unclear. As the Ras-Raf-Mek-Erk pathway communicates signals from the receptors on the cell surface into the cytoplasm and nucleus, it is reasonable to speculate that hyperactivation of Ras via Ras mutations lead to activation of the Raf-Mek-Erk pathway that may tune sensitivity to death. As post mitotic neurons have no Ras mutations, they may be less sensitive than cancer cells bearing this mutation.

3) The authors could simply state the IC50's for the different cells found in Figure 1 in the body of the text, rather than display the whole figure.

Figure 2 should show the vehicle alone pictures.

Response: We apologize for not making this clearer. We did not display the vehicle alone pictures in Figure 2 because of space restrictions. We have now included those pictures in Figure 1 including the dose-responses for erastin and HCA in PCN. We have also included the IC50s in Figures 1 and 14 (erastin and HCA dose-responses).

4) In Figures 2-5 I am not clear what the numbers in the table represent. Is there a better way to display them?

Response: We apologize again for the lack of clarity. The numbers show mean {plus minus} SD at representative concentration in brackets. We have exchanged the word “numbers” for “values” in the figure legend.

5) Are statistics based on each well in single experiments? If so, have the experiments been repeated?

Response: All data represent appropriately powered biological replicates. For MTT assay, each biological replicate is the mean of four technical replicates. We have clarified this in the Statistics section.

6) Figure 2: Actinomycin and UO126 images in PCN do not match survival numbers.

Response: We thank the reviewer for pointing this out to us. Images for U0126 match survival numbers. Please compare erastin + vehicle in Figure 1 in PCN to erastin + U0126 in Figure 2. For Actinomycin D, we have exchanged the image, which was inadvertently misplaced.

7) Figure 3: Baflomycin and Chloroquine images in PCN do not match survival numbers.

Response: Images for Bafilomycin match survival numbers. Please compare erastin + vehicle in Figure 1 in PCN to erastin + Bafilomycin in what is now Figure 4. For Chloroquine, we have exchanged the image which again was inadvertently misplaced.

8) Figures 6-8 should be combined into 1 figure highlighting the inhibitors that have a differential effect on normal vs. cancer cells.

Response: Figure 6-8 represented the complete dose-responses of all inhibitors in erastin-induced toxicity in HT1080 and erastin- and HCA-induced toxicity in PCNs. We have reorganized these figures (now Figure 3, 5, 7, 9) to match the previous Figure 2-5 (now Figures 2, 4, 6, 8).

9) Figure 2: “U0126” is written in the table and “U0124” in the figure.

Response: U0124 is the structural but inactive analog of U0126, we have used it as a pharmacological control for U0126. We have added U0124 to the table in Figure 2A. Similarly, we have used Necrostatin-1i as a negative control for Necrostatin-1 and have added Necrostatin-1i to the table in Figure 6A, and Nullscript for Scriptaid which was added to Figure 12.
