# Supplementary material for: Ferroptosis in Neurons and Cancer Cells Is Similar But Differentially Regulated by Histone Deacetylase Inhibitors
Source: eNeuro. 2019 Feb 15;6(1):ENEURO.0263-18.2019. doi: 10.1523/ENEURO.0263-18.2019 (PMC6378329; doi:10.1523/ENEURO.0263-18.2019)
Supplement: Figure 3-1 — Statistical data on ferroptosis inhibitors in HT1080 cells and primary cortical neurons. Download Figure 3-1, DOCX file. [file sup_enu-eN-NWR-0263-18-s02.docx]

**EXTENDED DATA FOR**

**Ferroptosis in neurons and cancer cells is similar but differentially regulated by histone deacetylase inhibitors**

**Detailed Statistical Analysis**

**Figure 3-1. Statistical data on ferroptosis inhibitors in HT1080 cells and primary cortical neurons**

| **Model** | **Cell Death Inhibitor** | **Kolmogorov-Smirnov test** | **Levené test** | **Omnibus Test** | **Posthoc Test** |
| --- | --- | --- | --- | --- | --- |
| **Erastin-induced toxicity in HT1080 cells** | **Actinomycin D** | Z=0.405, p=0.997 | F(4,40)=3.266, p=0.021 | Kruskal-Wallis test, χ²(4,N=45)=11.245, p=0.024, η²=0.256 | posthoc Mann-Whitney U with Bonferroni correction at α=0.0125: p=0.005 for 10nM, p=0.007 for 100nM |
|  | **Ferrostatin-1** | Z=0.913, p=0.376 | F(4,30)=0.786, p=0.568 | one-way ANOVA F(4,30)=19.472, p<0.001, partial-η²=0.764 | posthoc Bonferroni p<0.001 for 0.1-10µM |
|  | **Deferoxamine** | Z=0.806, p=0.535 | F(4,35)=1.593, p=0.198 | one-way ANOVA F(4,35)=23.022, p<0.001, partial-η²=0.725 | posthoc Bonferroni p<0.001 for 10-100µM |
|  | **N-Acetylcysteine** | Z=0.684, p=0.738 | F(4,25)=2.506, p=0.068 | one-way ANOVA F(4,25)=9.062, p<0.001, partial-η²=0.592 | posthoc Bonferroni p=0.005 for 250µM, p=0.001 for 500µM, p<0.001 for 1000µM |
|  | **Trolox, vitamin E analog** | Z=1.211, p=0.106 | F(4,25)=5.963, p=0.002 | Kruskal-Wallis test, χ²(4,N=30)=15.591, p=0.004, η²=0.538 | posthoc Mann-Whitney U with Bonferroni correction at α=0.0125: p=0.004 for 100µM |
|  | **U0126** | Z=0.830, p=0.497 | F(8,45)=1.995, p=0.069 | one-way ANOVA F(8,45)=15.628, p<0.001, partial-η²=0.735 | posthoc Bonferroni p<0.001 for 1-20µM U0126 vs. vehicle and for 1-5µM U0126 vs. the same concentration of U0124, p=0.009 for 10µM U0126 vs. 10µM U0124 |
| **Erastin-induced toxicity in primary cortial neurons** | **Actinomycin D** | Z=0.828, p=0.499 | F(4,25)=1.888, p=0.144 | one-way ANOVA F(4,25)=13.542, p<0.001, partial-η²=0.684 | posthoc Bonferroni p<0.001 for 1nM |
|  | **Ferrostatin-1** | Z=0.163, p=0.042 | F(4,25)=1.291, p=0.300 | Kruskal-Wallis test, χ²(4,N=30)=21.346, p<0.001, η²=0.736 | posthoc Mann-Whitney U with Bonferroni correction at α=0.0125: p=0.004 for 0.1-1µM |
|  | **Deferoxamine** | Z=0.120, p=0.067 | F(4,45)=7.141, p<0.001 | Kruskal-Wallis test, χ²(4,N=50)=15.019, p=0.005, η²=0.307 | posthoc Mann-Whitney U with Bonferroni correction at α=0.0125: p=0.007 for 25µM, p<0.001 for 50µM, p=0.001 for 100µM |
|  | **N-Acetylcysteine** | Z=0.192, p=0.019 | F(4,20)=3.200, p=0.035 | Kruskal-Wallis test, χ²(4,N=25)=18.292, p=0.001, η²=0.762 | posthoc Mann-Whitney U with Bonferroni correction at α=0.0125: p=0.009 for 10-100µM |
|  | **Trolox, vitamin E analog** | Z=0.142, p=0.042 | F(4,35)=3.320, p=0.021 | Kruskal-Wallis test, χ²(4,N=40)=22.787, p<0.001, η²=0.584 | posthoc Mann-Whitney U with Bonferroni correction at α=0.0125: p=0.001 for 100µM |
|  | **U0126** | Z=0.157, p=0.001 | F(8,54)=3.017, p=0.007 | Kruskal-Wallis test, χ²(8,N=63)=44.510, p<0.001, η²=0.718 | posthoc Mann-Whitney U with Bonferroni correction at α=0.0042: p=0.002 for 5-20µM U0126 vs. vehicle and vs. the same concentration of U0124 |
| **Glutamate analog (HCA)-induced toxicity in primary cortial neurons** | **Actinomycin D** | Z=0.897, p=0.397 | F(4,30)=2.313, p=0.080 | one-way ANOVA F(4,30)=2.151, p=0.099, partial-η²=0.223 | - |
|  | **Ferrostatin-1** | Z=0.253, p<0.001 | F(4,25)=5.905, p=0.002 | Kruskal-Wallis test, χ²(4,N=30)=21.567, p<0.001, η²=0.744 | posthoc Mann-Whitney U with Bonferroni correction at α=0.0125: p=0.004 for 0.1-1µM |
|  | **Deferoxamine** | Z=0.200, p=0.003 | F(4,25)=13.022, p<0.001 | Kruskal-Wallis test, χ²(4,N=30)=10.052, p=0.040, η²=0.347 | posthoc Mann-Whitney U with Bonferroni correction at α=0.0125: p=0.004 for 25µM, p=0.006 for 50µM |
|  | **N-Acetylcysteine** | Z=0.163, p=0.041 | F(4,25)=3.251, p=0.028 | Kruskal-Wallis test, χ²(4,N=30)=21.837, p<0.001, η²=0.753 | posthoc Mann-Whitney U with Bonferroni correction at α=0.0125: p=0.004 for 100-1000µM |
|  | **Trolox, vitamin E analog** | Z=0.109, p=0.200 | F(4,25)=2.373, p=0.080 | one-way ANOVA F(4,25)=11.482, p<0.001, partial-η²=0.648 | posthoc Bonferroni p<0.001 for 100µM |
|  | **U0126** | Z=0.213, p<0.001 | F(8,54)=17.269, p<0.001 | Kruskal-Wallis test, χ²(8,N=63)=45.544, p<0.001, η²=0.735 | posthoc Mann-Whitney U with Bonferroni correction at α=0.0042: p=0.002 for 5-20µM U0126 vs. vehicle and vs. the same concentration of U0124 |

**Figure 5-1. Statistical data on apoptosis inhibitors in HT1080 cells and primary cortical neurons**

| **Model** | **Cell Death Inhibitor** | **Kolmogorov-Smirnov test** | **Levené test** | **Omnibus Test** | **Posthoc Test** |
| --- | --- | --- | --- | --- | --- |
| **Erastin-induced toxicity in HT1080 cells** | **z-VAD-fmk** | Z=0.882, p=0.418 | F(4,30)=0.288, p=0.883 | one-way ANOVA F(4,30)=0.107, p=0.979, partial-η²=0.014 | - |
|  | **Cycloheximide** | Z=0.662, p=0.774 | F(4,45)=12.387, p<0.001 | Kruskal-Wallis test, χ²(4,N=50)=17.674, p=0.001, η²=0.361 | posthoc Mann-Whitney U with Bonferroni correction at α=0.0125: p<0.001 for 1µM, p=0.001 for 10µM, p=0.005 for 50µM |
|  | **Cyclosporin A** | Z=0.744, p=0.637 | F(4,20)=4.313, p=0.011 | Kruskal-Wallis test, χ²(4,N=25)=11.867, p=0.018, η²=0.494 | posthoc Mann-Whitney U with Bonferroni correction at α=0.0125: p=0.009 for 10µM |
|  | **SB203580** | Z=0.761, p=0.609 | F(4,15)=1.080, p=0.401 | one-way ANOVA F(4,15)=0.626, p=0.651, partial-η²=0.143 | - |
|  | **SP600125** | Z=0.780, p=0.577 | F(4,15)=4.060, p=0.020 | Kruskal-Wallis test, χ²(4,N=20)=0.914, p=0.923, η²=0.048 | - |
| **Erastin-induced toxicity in primary cortial neurons** | **z-VAD-fmk** | Z=0.963, p=0.311 | F(4,15)=1.572, p=0.233 | one-way ANOVA F(4,15)=1.184, p=0.358, partial-η²=0.240 | - |
|  | **Cycloheximide** | Z=0.157, p=0.114 | F(4,20)=2.263, p=0.098 | one-way ANOVA F(4,20)=2.499, p=0.075, partial-η²=0.333 | - |
|  | **Cyclosporin A** | Z=0.113, p=0.200 | F(4,30)=3.380, p=0.021 | Kruskal-Wallis test, χ²(4,N=35)=2.495, p=0.645, η²=0.073 | - |
|  | **SB203580** | Z=0.147, p=0.173 | F(4,20)=0.325, p=0.858 | one-way ANOVA F(4,20)=0.906, p=0.479, partial-η²=0.153 | - |
|  | **SP600125** | Z=0.114, p=0.200 | F(4,20)=0.059, p=0.993 | one-way ANOVA F(4,20)=1.180, p=0.350, partial-η²=0.191 | - |
| **Glutamate analog (HCA)-induced toxicity in primary cortial neurons** | **z-VAD-fmk** | Z=0.230, p=0.001 | F(4,20)=1.736, p=0.182 | Kruskal-Wallis test, χ²(4,N=25)=8.743, p=0.068, η²=0.364 | - |
|  | **Cycloheximide** | Z=0.151, p=0.200 | F(4,15)=0.527, p=0.718 | one-way ANOVA F(4,15)=9.544, p<0.001, partial-η²=0.718 | posthoc Bonferroni p=0.002 for 0.1µM |
|  | **Cyclosporin A** | Z=0.198, p<0.001 | F(4,40)=1.270, p=0.298 | Kruskal-Wallis test, χ²(4,N=45)=9.592, p=0.048, η²=0.218 | posthoc Mann-Whitney U with Bonferroni correction at α=0.0125: p=0.009 for 0.1µM and 5µM |
|  | **SB203580** | Z=1.524, p=0.019 | F(4,20)=8.341, p<0.001 | Kruskal-Wallis test, χ²(4,N=25)=12.236, p=0.016, η²=0.510 | posthoc Mann-Whitney U with Bonferroni correction at α=0.0125: p=0.009 for 5-30µM |
|  | **SP600125** | Z=1.575, p=0.014 | F(4,20)=7.557, p=0.001 | Kruskal-Wallis test, χ²(4,N=25)=12.025, p=0.017, η²=0.501 | posthoc Mann-Whitney U with Bonferroni correction at α=0.0125: p=0.009 for 1-5µM |

**Figure 7-1. Statistical data on parthanatos and necroptosis inhibitors in HT1080 cells and primary cortical neurons**

| **Model** | **Cell Death Inhibitor** | **Kolmogorov-Smirnov test** | **Levené test** | **Omnibus Test** | **Posthoc Test** |
| --- | --- | --- | --- | --- | --- |
| **Erastin-induced toxicity in HT1080 cells** | **DPQ, PARP inhibitor III** | Z=0.523, p=0.947 | F(4,15)=0.902, p=0.487 | one-way ANOVA F(4,15)=6.469, p=0.003, partial-η²=0.633 | posthoc Bonferroni p=0.007 for 50µM |
|  | **Olaparib (AZD-2281, trade name Lynparza)** | Z=0.498, p=0.965 | F(4,15)=1.222, p=0.343 | one-way ANOVA F(4,15)=1.249, p=0.333, partial-η²=0.250 | - |
|  | **GSK872** | Z=0.141, p<0.001 | F(4,80)=1.308, p=0.274 | Kruskal-Wallis test, χ²(4,N=85)=8.246, p=0.083, η²=0.098 | posthoc Mann-Whitney U with Bonferroni correction at α=0.0125: p=0.008 for 30µM |
|  | **Necrostatin-1** | Z=1.489, p=0.024 | F(8,72)=3.911, p=0.001 | Kruskal-Wallis test, χ²(4,N=81)=46.935, p<0.001, η²=0.587 | posthoc Mann-Whitney U with Bonferroni correction at α=0.0042: p=0.002 for 50µM Nec-1 vs. vehicle and vs. 50µM Nec-1i, p<0.001 for 100-250µM Nec-1 vs. vehicle and for 100µM Nec-1 vs. Nec-1i |
|  | **Necrosulfamide** | Z=0.507, p=0.959 | F(4,20)=11.367, p<0.001 | Kruskal-Wallis test, χ²(4,N=25)=19.857, p=0.001, η²=0.827 | posthoc Mann-Whitney U with Bonferroni correction at α=0.0125: p=0.009 for 1-5µM |
| **Erastin-induced toxicity in primary cortial neurons** | **DPQ, PARP inhibitor III** | Z=0.146, p=0.057 | F(4,30)=1.468, p=0.237 | one-way ANOVA F(4,30)=11.351, p<0.001, partial-η²=0.602 | posthoc Bonferroni p=0.001 for 50µM |
|  | **Olaparib (AZD-2281, trade name Lynparza)** | Z=0.134, p=0.180 | F(4,25)=0.130, p=0.970 | one-way ANOVA F(4,25)=3.243, p=0.028, partial-η²=0.342 | posthoc Bonferroni p>0.05 |
|  | **Necrostatin-1** | Z=0.904, p=0.387 | F(8,27)=1.727, p=0.137 | one-way ANOVA F(8,27)=7.135, p<0.001, partial-η²=0.679 | posthoc Bonferroni p=0.024 for 100µM Nec-1 vs. vehicle, p=0.008 for 250µM Nec-1 vs. vehicle, p=0.025 for 100µM Nec-1 vs. 100µM Nec-1i |
|  | **GSK872** | Z=0.121, p=0.143 | F(4,35)=0.381, p=0.821 | one-way ANOVA F(4,35)=4.382, p=0.006, partial-η²=0.334 | posthoc Bonferroni p=0.015 for 50µM |
|  | **Necrosulfamide** | Z=0.138, p=0.010 | F(4,50)=3.526, p=0.013 | Kruskal-Wallis test, χ²(4,N=55)=10.322, p=0.035, η²=0.191 | posthoc Mann-Whitney U with Bonferroni correction at α=0.0125: p=0.001 for 20µM |
| **Glutamate analog (HCA)-induced toxicity in primary cortial neurons** | **DPQ, PARP inhibitor III** | Z=0.163, p=0.170 | F(4,15)=0.739, p=0.580 | one-way ANOVA F(4,15)=6.984, p=0.002, partial-η²=0.651 | posthoc Bonferroni p=0.044 for 50µM |
|  | **Olaparib (AZD-2281, trade name Lynparza)** | Z=0.289, p=0.001 | F(4,10)=1.280, p=0.341 | Kruskal-Wallis test, χ²(4,N=15)=3.433, p=0.488, η²=0.245 | - |
|  | **Necrostatin-1** | Z=0.085, p=0.200 | F(8,46)=0.977, p=0.466 | one-way ANOVA F(8,46)=9.191, p<0.001, partial-η²=0.615 | posthoc Bonferroni p=0.001 for 250µM Nec-1 vs. vehicle, p=0.010 for 100µM Nec-1 vs. 100µM Nec-1i, p<0.001 for 250µM Nec-1 vs. 250µM Nec-1i |
|  | **GSK872** | Z=0.206, p=0.002 | F(4,25)=0.515, p=0.726 | Kruskal-Wallis test, χ²(4,N=30)=15.892, p=0.003, η²=0.548 | posthoc Mann-Whitney U with Bonferroni correction at α=0.0125: p=0.004 for 30µM |
|  | **Necrosulfamide** | Z=0.283, p<0.001 | F(4,55)=15.743, p<0.001 | Kruskal-Wallis test, χ²(4,N=60)=20.080, p<0.001, η²=0.340 | posthoc Mann-Whitney U with Bonferroni correction at α=0.0125: p=0.001 for 1µM and 20µM |

**Figure 9-1. Statistical data on autophagy inhibitors in HT1080 cells and primary cortical neurons**

| **Model** | **Cell Death Inhibitor** | **Kolmogorov-Smirnov test** | **Levené test** | **Omnibus Test** | **Posthoc Test** |
| --- | --- | --- | --- | --- | --- |
| **Erastin-induced toxicity in HT1080 cells** | **3-Methyladenine** | Z=0.899, p=0.394 | F(4,25)=2.375, p=0.079 | one-way ANOVA F(4,25)=2.450, p=0.072, partial-η²=0.282 | - |
|  | **Bafilomycin A1** | Z=0.967, p=0.307 | F(4,35)=2.317, p=0.076 | one-way ANOVA F(4,35)=18.063, p<0.001, partial-η²=0.680 | posthoc Bonferroni p<0.001 for 10-100nM |
|  | **Chloroquine diphosphate salt** | Z=1.054, p=0.217 | F(4,30)=1.951, p=0.128 | one-way ANOVA F(4,30)=33.831, p<0.001, partial-η²=0.819 | posthoc Bonferroni p<0.001 for 10µM |
|  | **Rapamycin** | Z=0.852, p=0.463 | F(4,55)=0.302, p=0.875 | one-way ANOVA F(4,55)=10.256, p<0.001, partial-η²=0.427 | posthoc Bonferroni p<0.001 for 0.1-1µM, p=0.005 for 5µM |
|  | **Mitochondrial division inhibitor 1** | Z=0.612, p=0.847 | F(4,25)=1.326, p=0.288 | one-way ANOVA F(4,25)=5.615, p=0.002, partial-η²=0.473 | posthoc Bonferroni p>0.05 |
| **Erastin-induced toxicity in primary cortial neurons** | **3-Methyladenine** | Z=0.171, p=0.005 | F(4,35)=2.219, p=0.087 | Kruskal-Wallis test, χ²(4,N=40)=7.537, p=0.110, η²=0.193 | - |
|  | **Bafilomycin A1** | Z=0.656, p=0.782 | F(4,30)=3.174, p=0.027 | Kruskal-Wallis test, χ²(4,N=35)=6.585, p=0.160, η²=0.194 | - |
|  | **Chloroquine diphosphate salt** | Z=0.165, p=0.017 | F(4,30)=1.244, p=0.313 | Kruskal-Wallis test, χ²(4,N=35)=3.361, p=0.499, η²=0.099 | - |
|  | **Rapamycin** | Z=0.147, p=0.099 | F(4,25)=0.459, p=0.765 | one-way ANOVA F(4,25)=12.875, p<0.001, partial-η²=0.673 | posthoc Bonferroni p<0.001 for 0.1-5µM |
|  | **Mitochondrial division inhibitor 1** | Z=0.137, p=0.093 | F(4,30)=0.481, p=0.750 | one-way ANOVA F(4,30)=4.435, p=0.006, partial-η²=0.372 | posthoc Bonferroni p>0.05 |
| **Glutamate analog (HCA)-induced toxicity in primary cortial neurons** | **3-Methyladenine** | Z=0.155, p=0.002 | F(4,50)=7.294, p<0.001 | Kruskal-Wallis test, χ²(4,N=55)=31.122, p<0.001, η²=0.576 | posthoc Mann-Whitney U with Bonferroni correction at α=0.0125: p=0.002 for 0.1mM, p<0.001 for 1.5mM |
|  | **Bafilomycin A1** | Z=0.844, p=0.475 | F(4,35)=4.441, p=0.005 | Kruskal-Wallis test, χ²(4,N=40)=20.285, p<0.001, η²=0.520 | posthoc Mann-Whitney U with Bonferroni correction at α=0.0125: p=0.001 for 1nM, p=0.005 for 50nM |
|  | **Chloroquine diphosphate salt** | Z=0.201, p=0.003 | F(4,25)=5.736, p=0.002 | Kruskal-Wallis test, χ²(4,N=30)=7.826, p=0.098, η²=0.270 | - |
|  | **Rapamycin** | Z=0.167, p=0.015 | F(4,30)=0.579, p=0.680 | Kruskal-Wallis test, χ²(4,N=35)=19.701, p=0.001, η²=0.579 | posthoc Mann-Whitney U with Bonferroni correction at α=0.0125: p=0.002 for 0.1-5µM |
|  | **Mitochondrial division inhibitor 1** | Z=0.236, p<0.001 | F(4,35)=3.497, p=0.017 | Kruskal-Wallis test, χ²(4,N=40)=24.531, p<0.001, η²=0.629 | posthoc Mann-Whitney U with Bonferroni correction at α=0.0125: p=0.005 for 0.1µM, p=0.009 for 1µM |

**Figure 10-1: Statistical data on levels of pRIP1 in erastin- and glutamate analog (HCA)-induced cell death**

|  | **Kolmogorov-Smirnov test** | **Levené test** | **Omnibus Test** | **Posthoc Test** |
| --- | --- | --- | --- | --- |
| **pRIP1 in erastin** | Z=2.623, p<0.001 | F(7,56)=45.213, p<0.001 | Kruskal-Wallis test, χ²(7,N=64)=45.028, p<0.001, η²=0.715 | posthoc Mann-Whitney U with Bonferroni correction at α=0.0056: p<0.001 for 8-12 hours erastin and 8 hours TNFα/zVAD vs. 0 hours, p=0.006 for 8 hours TNFα/zVAD + Nec-1 vs. 8 hours TNFα/zVAD |
| **pRIP1 in HCA** | Z=2.138, p<0.001 | F(7,40)=6.337, p<0.001 | Kruskal-Wallis test, χ²(7,N=48)=36.378, p<0.001, η²=0.774 | posthoc Mann-Whitney U with Bonferroni correction at α=0.0056: p=0.002 for 12 hours HCA and 8 hours TNFα/zVAD vs. 0 hours, p=0.004 for 8 hours TNFα/zVAD + Nec-1 vs. 8 hours TNFα/zVAD |

**Figure 13-1. Statistical data on cell death inhibitors in erastin-induced cell death in HT1080 cells**

| **Cell Death Inhibitor** | **Kolmogorov-Smirnov test** | **Levené test** | **Omnibus Test** | **Posthoc Test** |
| --- | --- | --- | --- | --- |
| **Adaptaquin** | Z=0.682, p=0.740 | F(4,20)=4.566, p=0.009 | Kruskal-Wallis test, χ²(4,N=25)=14.201, p=0.007, η²=0.592 | posthoc Mann-Whitney U with Bonferroni correction at α=0.0125: p=0.009 for 0.5-1µM |
| **Mithramycin** | Z=0.124, p=0.200 | F(4,20)=0.217, p=0.926 | one-way ANOVA F(4,20)=6.379, p=0.002, partial-η²=0.561 | posthoc Bonferroni p=0.006 for 50nM, p=0.003 for 100nM, p=0.015 for 200nM, p=0.020 for 300nM |
| **Cystamine** | Z=0.889, p=0.408 | F(4,35)=1.040, p=0.401 | one-way ANOVA F(4,35)=30.390, p<0.001, partial-η²=0.776 | posthoc Bonferroni p<0.001 for 10-100µM |
| **B003** | Z=0.894, p=0.401 | F(4,25)=1.374, p=0.271 | one-way ANOVA F(4,25)=20.840, p<0.001, partial-η²=0.769 | posthoc Bonferroni p=0.017 for 100µM , p<0.001 for 200µM |
| **D004** | Z=0.143, p=0.069 | F(4,30)=0.089, p=0.985 | one-way ANOVA F(4,30)=0.482, p=0.748, partial-η²=0.060 | - |
| **Apicidin** | Z=0.200, p=0.003 | F(4,25)=0.749, p=0.568 | Kruskal-Wallis test, χ²(4,N=30)=20.830, p<0.001, η²=0.718 | posthoc Mann-Whitney U with Bonferroni correction at α=0.0125: p=0.010 for 10nM, p=0.004 for 50-500nM |
| **Butyrate** | Z=0.108, p=0.098 | F(7,48)=1.208, p=0.317 | one-way ANOVA F(7,48)=3.534, p=0.004, partial-η²=0.340 | posthoc Bonferroni p=0.009 for 2.5mM, p=0.006 for 5mM, p=0.005 for 7.5mM |
| **MS-275** | Z=0.155, p=0.065 | F(4,25)=0.305, p=0.872 | one-way ANOVA F(4,25)=8.381, p<0.001, partial-η²=0.573 | posthoc Bonferroni p=0.017 for 0.5µM, p=0.015 for 1µM |
| **Scriptaid** | Z=1.196, p=0.115 | F(8,54)=2.179, p=0.044 | Kruskal-Wallis test, χ²(8,N=63)=26.183, p=0.001, η²=0.422 | posthoc Mann-Whitney U with Bonferroni correction at α=0.0042: p>0.0042 |

**Figure 13-2. Statistical data on gene expression after mithramycin treatment in HT1080 cells**

|  | **Kolmogorov-Smirnov test** | **Levené test** | **Omnibus Test** | **Posthoc Test** |
| --- | --- | --- | --- | --- |
| **c-Myc** | Z=0.203, p=0.097 | F(2,12)=15.577, p<0.001 | Kruskal-Wallis test, χ²(2,N=15)=0.181, p=0.913, η²=0.013 | - |
| **p21** | Z=0.366, p<0.001 | F(2,12)=4.816, p=0.029 | Kruskal-Wallis test, χ²(2,N=15)=12.522, p=0.002, η²=0.894 | posthoc Mann-Whitney U with Bonferroni correction at α=0.025: p=0.009 for 50 and 300nM vs. vehicle |

**Figure 13-3. Statistical data on Scriptaid and Nullscript in erastin-induced death in PCN**

| **Cell Death Inhibitor** | **Kolmogorov-Smirnov test** | **Levené test** | **Omnibus Test** | **Posthoc Test** |
| --- | --- | --- | --- | --- |
| **Scriptaid** | Z=0.099, p=0.200 | F(8,18)=2.482, p=0.052 | one-way ANOVA F(8,18)=6.589, p<0.001, partial-η²=0.745 | posthoc Bonferroni p=0.036 for 0.5-1µM Scriptaid vs. vehicle, p=0.002 for 1µM Scriptaid vs. 1µM Nullscript |

**Figure 13-4. Statistical data on HDAC gene expression in PCN versus HT1080 cells**

| **Kolmogorov-Smirnov test** | **Levené test** | **Omnibus Test** | **Posthoc Test** |
| --- | --- | --- | --- |
| Z=0.155, p=0.005 | F(7,40)=13.625, p<0.001 | Kruskal-Wallis test, χ²(7,N=48)=41.414, p<0.001, η²=0.881 | posthoc Mann-Whitney U with Bonferroni correction at α=0.0125: p=0.002 for HDAC 1 and p=0.004 for HDAC 8 PCN vs. HT1080 |

**Figure 14-1. Statistical data on Scriptaid in erastin-induced cell death in SH-SY5Y and Hep3B cells**

| **Cell line** | **Kolmogorov-Smirnov test** | **Levené test** | **Omnibus test** | **Posthoc test** |
| --- | --- | --- | --- | --- |
| **SH-SY5Y** | Z=0.116, p=0.200 | F(8,21)=0.482, p=0.855 | one-way ANOVA F(8,21)=9.387, p<0.001, partial-η²=0.781 | posthoc Bonferroni p=0.008 for 2.5µM, p=0.001 for 5µM, p<0.001 for 10µM Scriptaid vs. vehicle, p=0.042 for 2.5µM, p=0.030 for 5µM Scriptaid vs. the same concentration of Nullscript |
| **Hep3B** | Z=0.137, p=0.155 | F(8,21)=0.911, p=0.526 | one-way ANOVA F(8,21)=14.499, p<0.001, partial-η²=0.847 | posthoc Bonferroni p<0.001 for 10µM Scriptaid vs. vehicle |
